# Adaptive grid resilient based protection method for multi fault scenarios in medium voltage quintuple DC microgrid system

**DOI:** 10.1038/s41598-025-88833-4

**Published:** 2025-02-12

**Authors:** S. Faazila Fathima, L. Premalatha

**Affiliations:** School of Electrical Engineering, Vellore Institute of Technology, Chennai, Tamil Nadu 600127 India

**Keywords:** Bidirectional Dial’s, Fault detection, Fault tripping time, Level order tree traversal (LOTT), Smart Power Relay (SPR), Quintuple DC microgrid, Electrical and electronic engineering, Solar energy, Wind energy

## Abstract

Multi-microgrid systems offer a versatile solution to many of the challenges including issues on power glitches, grid flow optimization, stability and protection system malfunction faced by traditional centralized power grids. By enhancing resilience, integrating renewable energy, improving efficiency, and supporting economic and environmental goals, they represent a forward-looking approach to modern energy management. Despite of technological progress in fault current detection, significant challenges oriented to false tripping and protection blinding in multi- microgrid structures compared to single microgrid topology still remain unresolved. The primary aspects that require focus while multigrid resilience includes fast fault detection and interruption, nuisance tripping and blinding of protection. This paper enhances the fault isolation speed by proposing adaptive grid resilient scheme (AGRS) with fault identification method using level order tree traversal (LOTT) and Bidirectional Dial’s algorithm, which functions with the assistance of MGMFDS (Microgrid Monitoring Fault Detection System). The presented work analyses the implementation of the proposed adaptive algorithm and tested for various types of faults in a 35-bus quintuple system, where its efficacy in interrupting the fault takes 2.64 ms. The experiments for the proposed work are conducted using a real time simulator for model in loop (MIL) and control hardware in loop (CHIL) testing.

**Supplementary Information:**

The online version contains supplementary material available at 10.1038/s41598-025-88833-4.

## Introduction

The escalating need for DC powered devices across industrial, commercial, and residential sectors has led to a significant existence of renewable energy sources (RES) in today’s power systems. Compared to AC microgrids, DC microgrids excel in terms of steadfastness, flexibility, efficacy, and resilience^1^. DC microgrids are increasingly incorporating many electrical components because they eliminate the power factor and frequency issues typically encountered in AC systems, while also providing greater efficiency and reducing the number of conversion processes. However, maintaining the reliability of DC microgrids necessitates a responsive and precise protection system due to the direct current’s non-cyclic nature and its dynamics. Protection challenges include controlling fault current levels, preventing false tripping, avoiding protection device impairment, and prohibiting automatic and uncoordinated breaker reclosing. Dual-direction protection device have emerged as solutions to safeguard DC electronic devices and renewable energy sources connected to the grid from the above mentioned challenges^2^.

In DC grids, directional overcurrent (OC) safeguarding is commonly applied and essential for promptly clearing faults. Synchronizing protection for directional OC relays is difficult due to the necessity for quick response to the extremely rapid rise times of fault currents in DC systems. During faults in DC microgrids, aligning downstream and upstream relays within a very limited timeframe is crucial. In a well-organized protection arrangement, the downstream relay should initiate a trip signal if both relays identify a fault, while the upstream relay should refrain from triggering, ensuring strong selectivity and preventing unwanted outages. Various OC relay characteristics, each incorporating delays to ensure selective action and reliable protection, are employed to coordinate their operation, and by considering these characteristics along with the dynamics of distributed generation, loading the relay working conditions as Python scripts in the protection controller helps avoid false tripping and blinding of protection^3^.

**Fig. 1:**
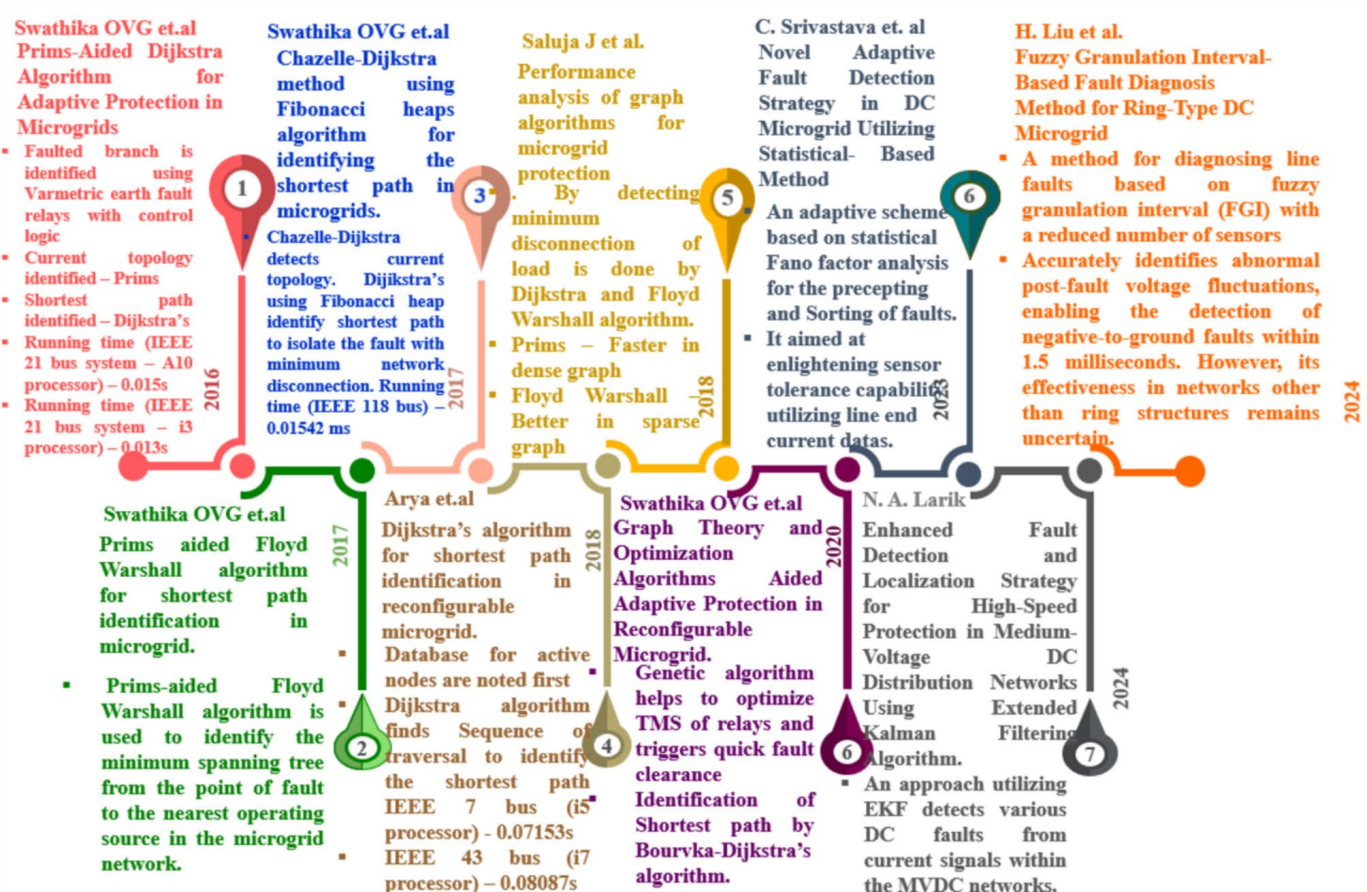
Recent literature on adaptive protection of microgrid.

Effective DC Circuit Breaker (DCCB) topologies are essential to quickly suppress faults and manage them without disrupting integrated renewables. Though there are many breakers, breaker interfacing with communication technology is infancy. Implementing adaptive algorithms in microgrids enhances grid resilience and accelerates fault isolation. Among various technologies, active bus identification combined with shortest path detection is crucial for fault detection and suppression. In^4^, Prim’s algorithm distinguishes between operational and non-operational buses within the network, while Dijkstra calculates the minimum distance route from the faulted location to the closest distributed generation (DG), with this heuristic method being validated using IEEE 21-bus and 40-bus microgrid systems. A comparable approach is investigated in^5^, where the Fenwick algorithm identifies operational buses and bidirectional Dijkstra’s algorithm determines the most efficient route until the optimal route to the nearest operational DG is found. These advancements underscore the importance of robust protection mechanisms and adaptive algorithms in ensuring the reliability and efficiency of DC microgrids in modern power systems and the literature as like [4&5] in protecting the microgrid using graph algorithm are shown in Fig. 1.

In^6^, Chazelle detects the operating and non-operating buses, and the Dijkstra algorithm finds the minimal distance path from the fault point to the nearest operating distributed generation, while in^7^, a similar approach combines Prim’s with the Floyd-Warshall algorithm in the microgrid network to determine the operating and non-operating buses and the most efficient route from the fault to the DG, exploring all potential paths between each pair of vertices and selecting the optimal route. A similar methodology is applied in^8^, where a radial structure is used in the proposed graph algorithm to ascertain the minimal distance route from the fault point to the point of common coupling (PCC), with active nodes identified using both Kruskal’s and Prim’s algorithms. Effective fault identification with minimal network reconfiguration is realised using Dijkstra’s and the Floyd-Warshall algorithms. This study concludes that Prim’s algorithm executes proficiently in impenetrable graphs, while the Floyd-Warshall algorithm is more suitable for sparse graphs. In^9^, active bus data is initially recorded, and the sequence of traversal to determine the minimal distance is tested on IEEE 7-bus and IEEE 43-bus systems using i5 and i7 processors, with fault detection in the IEEE 7-bus system taking 0.07153s on an i5 processor and in the IEEE 43-bus system taking 0.08087s on an i7 processor. In^10^, an adaptive protection scheme is proposed to minimize network disconnection during faults, utilizing genetic algorithms to optimize the Time Multiplier Setting (TMS) of relays and ensure rapid fault clearance while the most efficient route is recognised by Bourvka-Dijkstra algorithm.

An IED based relay coordination using goose protocol in^11^helps to communicate between the relays to avoid false tripping scenario. With K means clustering methodology and communication speed of 100 Mbps, the GOOSE message transfer delay is with a minimal value of 0.066 s resulting in detecting and responding the fault within 0.5ms. But, the usage of this methodology needs to be validated for MVDC and HVDC applications to assess its rapidity and sensitivity in protection. A 500 V ring DC bus configuration is proposed in^12^with a novel technique that is suggested for voltage travelling wave with DWT coefficient where the energy is subsequently assessed, and if it surpasses a predetermined threshold, results in identifying the fault and the validation of this methodology is carried out in real time simulator without any over runs and time delays. A digital signal processing approach based on empirical mode decomposition and Hilbert transform^13^ examines the fault current transients and rapid fault detection through the application of instantaneous frequency and time-frequency-magnitude spectrum analysis. This approach does not validate for simultaneous fault occurrence to prove the rigidity of the system.

In^25^, for the deployment of MVDC shipboard systems, the protection system monitoring is segmented into separate zones to protect the system using Linear Parameter Varying (LPV)-Unknown Input Observers (UIOs) to detect and resolve multiple faults with considerable computational complexity. A protection framework based on an ensemble classifier^26^has been proposed for DC microgrids to assess the network configuration in response to varying solar irradiance and wind speed. This method leverages local data for fault detection, topology recognition, fault identification, and classification via a random subspace sampling-based ensemble classifier technique. A variance-driven approach^27^is employed, where variations in input data under different fault scenarios are examined using PSCAD. In this technique, forward and reverse faults are detected based on the Difference of Mean (DoM) between consecutive windows, along with the standard Inverse Definite Minimum Time (IDMT) relay characteristics defined in the IEC 60255-151standards. The relay settings are determined by formulating a non-linear optimization problem, ensuring that the primary relay sends the tripping command within 1 millisecond. In^28^, a resilient-based methodology, considering the dynamics of renewable energy, determines network mode identification, fault detection/classification (CNN-based protection), segment forecasting, and location assessment using linear discriminant analysis (LDA), support vector machine (SVM), and decision tree (DT)-based algorithms, tested under various microgrid conditions with a free-link communication approach. Recent grounding methodologies using sequentially-switched grounding connections and current adjoint index [29 & 32], along with recent studies [30 & 31] incorporating Support Vector Machines and Bagged Trees in Machine Learning techniques, and Real-Time Wavelet Analysis methods, have provided solutions for fault detection, classification, and location in microgrids.

The literature reviewed thus far addresses specific configurations for which the proposed methods are intended, yet significant uncertainty remains regarding their applicability to various DC configurations, highlighting the need for an adaptable approach that can accommodate all types of DC microgrid configurations. Consequently, given the dependency of the protection system for Distributed Control and Management Grid (DCMG) on specific configurations, it is imperative to develop a suitable protection scheme that can be universally applied across all system configurations. Thus, this proposal proposes a smart power relay (SPR) integration based on implementation purpose and location, and a failure zone detection method employing a LOTT algorithm with segment tree functions expressed in Python scripts in the protection controller. The segment tree function updates an array with local parameters and fixes the relay minimum and maximum values as threshold fixation. The SPR can be implemented at the two ends of transmission line where one relay identifies the current direction and the other relay works based on overcurrent principle. This system tracks changes in power and voltage profiles over time, monitored by a protection controller, with the values and sudden load changes stored in a lookup table (LUT) based on normal and abnormal operating conditions, ensuring that load variations are not false evaluated for fault occurrences. The purpose of this paper is to resolve the issues in protecting DC microgrids with adaptive resilient based system modelling.

The innovative aspects of this paper are presented below.

1. With overcurrent data assessed by smart power relay (SPR) in conjunction with the protection controller, a resilient based method is proposed that uses (i) LOTT algorithm to find the operating bus (OB), Dormant bus (DB), and live transmission line and (ii) bidirectional Dial’s algorithm to compute the optimal route from the faulted location to the closest DG.
2. This advanced featured technique is implemented in a DC microgrid and validated for a several faults with segment tree functions, including simultaneous fault (SF), reverse fault (RF), and multi-faults (MF).
3. The results are validated in MIL and CHIL testing through a real-time simulation environment, considering the DG dynamics.

The paper is organized as follows: The seven-bus quintuple DC microgrid modelling is briefly described in Section II. The LOTT algorithm with the overcurrent detection premise is used to describe fault detection in Section III. Section IV examines the outcomes of the suggested work. Section V provides a conclusion of the findings and future works.

## Vindication of the proposed work

### Requisite for OC protection to determine the operating bus and minimal distant path

To improve the precision of fault location detection and reduce interrupting time, it is essential to identify the operating bus (OB) and dormant buses (DB), and to identify the quickest route from the fault location to the nearest distributed generation source. Monitoring local parameters such as current and power, and forwarding these datas to the protection controller, facilitates the implementation of advanced graph algorithms which further aids in optimizing fault location and thereby improving system efficiency, ultimately leading to more resilient operations and sustainable power delivery to consumers.

### Peculiarity of adaptive fault detection

In the proposed work, the enhanced graph algorithms for level-based protection are implemented for determining OB, DB and minimal path from fault location as it has an enhanced feature that accumulates data from different segments in the network. The level-based approach involves determining faults according to hierarchical importance, based on the type of connection interfaced with the bus, such as connections to loads or generation sources. The primary level preference for protection is made for PCC/ common bus in which all DCMGs are interfaced and the bus connected with DG’s and loads are in secondary level of protection where the datas from SPR helps the LOTT to easily predict the OB and DB, thereby the fault location can be predicted easily and the minimal distance path from the fault location to the proximally located DG is identified by bidirectional Dial’s algorithm. Unlike existing literature on protection strategies for DC microgrids which does not overlook on simultaneous fault and multi-fault scenarios, this study pioneers in identifying and interrupting all kinds of faults in DC microgrid configurations. Likewise, it employs the bidirectional Dial’s algorithm to identify the shortest distance from a fault to the closest distributed generation, considering reverse fault currents influenced by dominant distributed generation (DG) as per the LOTT algorithm’s recommendations. The voltage, current and power are noted, thereby the SPR checks for +/- 10% of the rated voltage, 40% of rated current and 10% of rated power that predicts the upper and lower threshold with respect to time and this research aids in achieving Sustainable Development Goal 7 in the context of smart grids, facilitating global access to affordable, reliable, and sustainable energy solutions.

## Modelling of 35 bus quintuple DC microgrid system

**Fig. 2:**
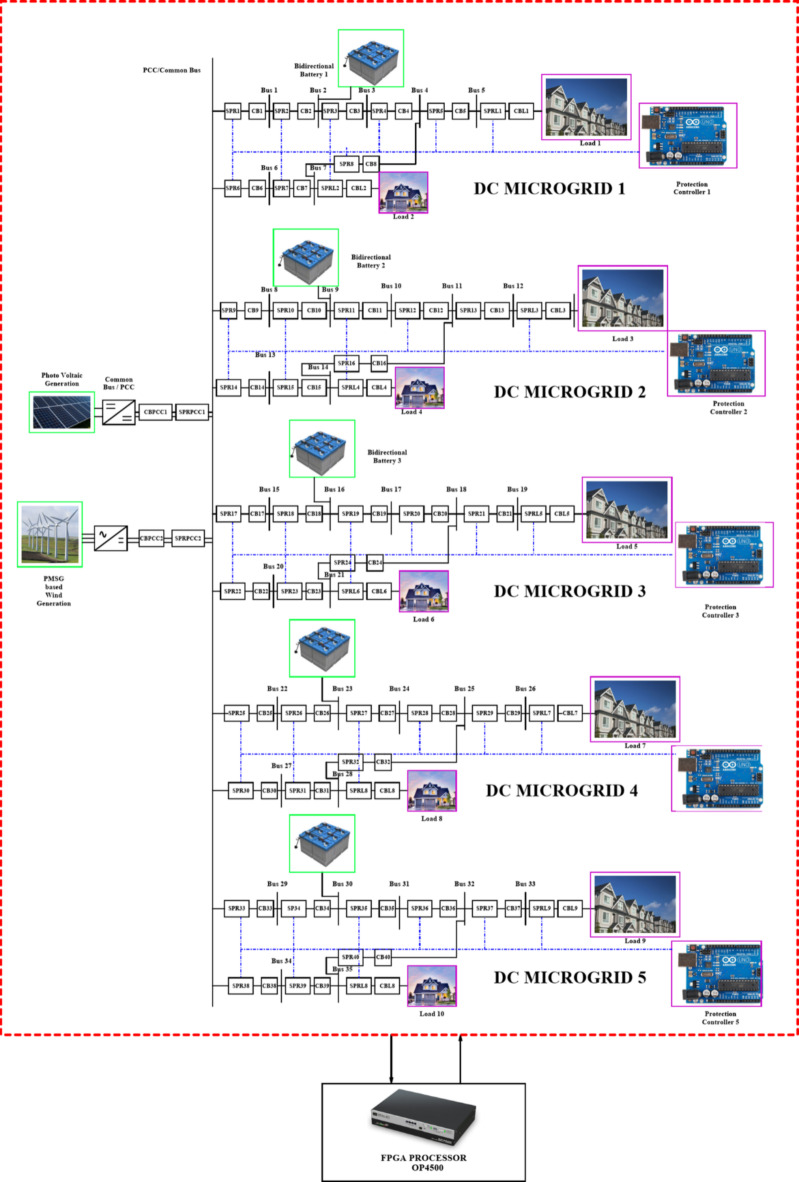
Quintuple multi-DC microgrid system.

The adaptive network is modelled with a self-governing microgrid functions solely where each microgrid interfaced 1500 V DC bus where the DC voltage is regulated by DC link capacitor and maintains the voltage at DC bus. Each DC microgrid modelling is based on the parameters mentioned in Table 1^10^. In this article, the renewable resources taken into considerations are a bidirectional battery, a 50 kW PV and a 45 kW Permanent Magnet synchronous generator-based wind turbine and thereby the radial configuration of quintuple DC microgrid architecture is shown in Fig. 2. The Photovoltaic (PV) array in DC microgrid is to expedite the real-time system thereby adding up the series PV panels provides higher voltage, whereas parallel interfaced PV panels deliver higher current. The solar irradiance level taken for consideration is 1000 W/m^2^ and the PV basic module is structured in relevant to Shockley diode equation.

**Table 1:** Parameters with ratings considered for Quintuple DCMG [16&17].

Parameters﻿	Ratings
System Voltage	1500 V
DC Capacity DCMG 1,2,3 & 4 DCMG 5	100 kW 1500 V, 15 A (each) 1500 V, 6 A
Battery Voltage, Ampere hour	14.2 V, 35Ah.
PV Generation	50 kW
PV Irradiation Level	1000 W/m^2^
Optimum temperature	25^o^C
Modules in series & parallel	10*5
PMSG based WECS	45 kW
β	0^o^
WV_WT_	30 m/s
Transmission line distance	2 km
Line Resistance	222mΩ/km
Line Inductance	0.99mH/km
Line Capacitance	22µF/km
Switching Frequency	25 kHz
LC Filter	49 uH and 2200 uF

The photo-current and saturation current of PV,

1 $$\:{I}_{ph}=\:\left[{I}_{scr}+\:{K}_{i}\:\left(T-298\right)*?/1000\right]$$

2 $$\:{I}_{rs}=\:{I}_{scr}/\:\left[\text{exp}\left(\frac{q{V}_{oc}}{{N}_{s}kAT}\right)-1\right]$$

The current output of PV module for IPV which can be written as,

3 $$\:{I}_{PV}={N}_{p}*{I}_{ph}-{N}_{p}*{I}_{o}\left[\text{exp}\left\{q*\frac{{V}_{PV}+{I}_{PV}{R}_{s}}{{N}_{s}AkT}\right\}-1\right]$$

Based on voltage and current requirements, the proposed PV array comprises PV modules integrated to achieve an output voltage of 1500 V. The solar irradiance is maintained at 1000 W/m², and the cell temperature is 25 °C^16^. A PMSG fixed speed is used as it does not need external excitation because the magnet or electromagnet attached to the stator supplies the necessary excitation. The wind turbine is modelled with 30 m/s and taking the pitch angle as 0^o^, the aerodynamic model can be written as,

=1/2()^3^ (4).

where P is Power generated by wind, ρ mentions as air density, A is blade area, v mentions as wind speed, C_p_ as power coefficient and λ as tip speed ratio.

=/30 (5).

where n is Rotor rotational speed of the wind turbine in revolutions per minute (rpm).

T_m_ = P_ω_ / ω_m_ = {0.5 ρ A_r_ C_p_(α,β) v^3^ }/ ω_m_ (6).

C_p_ (β, λ) = C_1_{(C_2_/z - C_3_β - C_4_) e^-C^_5_^/z^ + C_6_ λ (7).

A rectifier then converts the AC output from the turbine into DC, using an LC filter with values of 47 µH and 220 µF. Battery modeling is essential because of the bidirectional power flow in the microgrid. Consequently, a bidirectional battery model with a 35 Ah capacity, paired with a buck-boost converter, is necessary for the DC bus and it consists of a lithium battery connected to a bidirectional buck-boost DC-DC converter^17^. When the power generated by Distributed Generators (DGs) is insufficient to meet the load demands, the BS compensates for the deficit and stabilizes the voltage. On the other hand, when the power generated exceeds the load demand, the BS enters charging mode and with the following equation, the relationship between the BS and interfaced converter are described.

R_c_C_c_ (dv_ibb_/dt) + V_ibb_ = V_EMF_ – R_Ω_ R_C_C_C_ (di_ibb_/dt) – (R_Ω_ R_C_C_C_) i_ibb_ (8).

where R_C_ and C_C_ are resistor and capacitor during diffusion process, R_Ω_ is series resistance and V_EMF_ is the open circuit voltage, V_ibb_ and i_ibb_ represent the voltage and current of the input terminals of the buck-boost converter. Each DC Microgrid (DCMG 1, 2, 3 and 4) works with the resistive load of 100 Ω and DCMG 5 works with the resistive load of 250 Ω respectively.

**Fig. 3:**
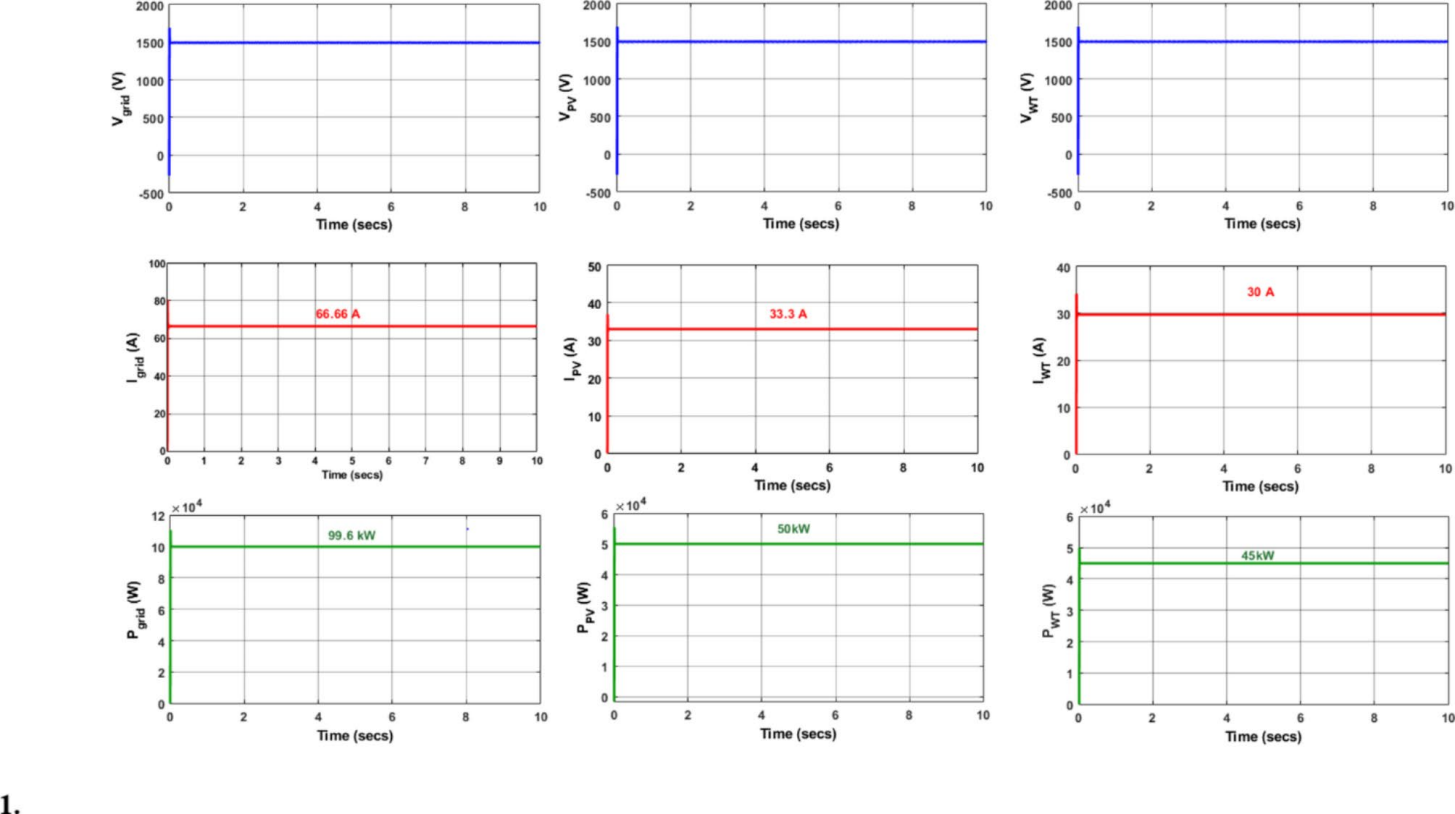
(**a**) DC grid voltage (**b**) PV voltage (**c**) Wind turbine interfaced converter voltage (**d**) DC grid current (**e**) PV current (**f**) Wind turbine interfaced converter current (**g**) DC grid power, (**h**) PV grid power (**i**) Power generated by Wind turbine interfaced converter.

The Fig. 3 illustrates the voltage, current and power of PV, wind turbine and DC grid and the Table 1provides the specification of renewables with ratings considered for each microgrid^10^ in Quintuple structure [16&17] respectively.

The relay and DC breaker may malfunction with conventional algorithm as the relay status monitored by the controller for large network needs to be addressed evidently to avoid the false tripping and protection blinding. As the conventional algorithm takes longer duration to determine the fault, a rapid relay interaction that works in a novel algorithm adapting the DC microgrid network is required to prove the swiftness in fault detection. Thus, the LOTT algorithm in united with bidirectional Dial’s provides rapidity in locating and determining the faulted point to distributed generation.

## Working of LOTT and Bidirectional Dial’s algorithm

### LOTT algorithm

An adaptive (LOTT) algorithm- based overcurrent protection technique detects the OB and DB during abnormal operating conditions. The protection controller operates according to the programmed instructions it receives, and the OB are identified by level order tree traversal (LOTT) algorithm. It predetermines the OB and configuration and if there are any presence of DB, the protection controller saves the information and tries to find the faulted branch from the local measurements using LOTT algorithm. This helps in identifying the faulted branch and helps in tripping the corresponding DCCB interrupts the fault and provides faster restoration.

1. Create an empty queue Q.
2. Enqueue the root node of the tree to Q.
3. Loop while Q is not empty.
  - Dequeue a node from Q and visit it.
  - Enqueue the left child of the dequeued node if it exists.
  - Enqueue the right child of the dequeued node if it exists.

### Bidirectional Dial’s algorithm

Dijkstra’s algorithm is slower than Dial’s algorithm for a DC microgrid with small-distance DC transmission lines, and its time complexity is O(n + wm), where ‘w’ represents the distance between buses, ‘n’ is the number of buses, and ‘m’ is the number of DC transmission lines between buses. Dial’s algorithm stores the values of distance, the number of buses, the number of DC transmission lines between buses, voltage, current, and power profiles in a bucket, which is similar to stacks in data structure algorithms.

The steps followed in Bidirectional Dial’s algorithm are:

4. Initialize a set of buckets or stacks to store the values considered, where each bucket contains the distance between all buses with respect to a common bus, which estimates the current shortest path.
5. Start with the source bus (common bus) and set its initial shortest path estimate to 0.
6. For each bus adjacent to the common bus, add it to the bucket corresponding to its distance from the common bus.
7. If the shortest path estimate from the previous calculation is greater than the new estimation, update the values and move it to the corresponding bucket.
8. Return the shortest path estimates for all nodes.

The concept behind Dial’s algorithm is to optimize the iterative process of Dijkstra’s algorithm by avoiding the need to scan all nodes at each iteration. Instead, the group the nodes are fed into buckets according to their shortest path estimate and only process the nodes with the smallest estimate in each iteration. This approach reduces the number of nodes that require processing, resulting in a faster algorithm, particularly when dealing with a small range of edge weights. Therefore, the bidirectional dial’s algorithm finds the distance between the accurate fault location to proximal operating distributed generation.

### Working of proposed graph algorithm in Quintuple DC microgrid system

**Fig. 4:**
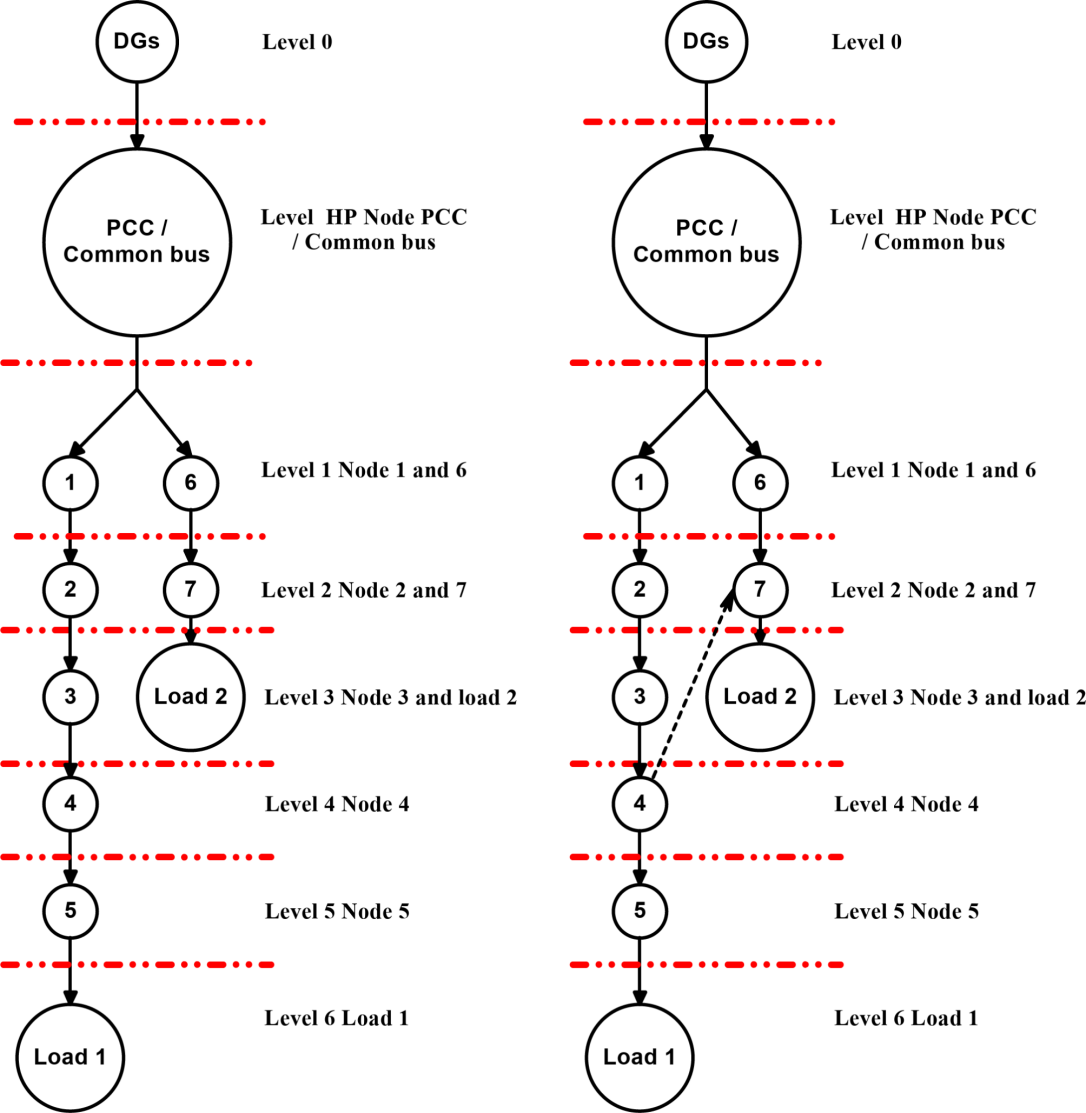
Working of LOTT in DCMG 1 configuration (**a**) Normal mode (**b**) Reconfiguration mode.

The Fig. 4exploits the function of LOTT algorithm in DC microgrid 1 for normal mode and reconfiguration mode of operation. The high priority (HP) level is the primary level of protection given to PCC/ common bus as it is interfaced with renewables and DC microgrid segments. The MGMFDS (Microgrid Monitoring Fault Detection System) integrated with LOTT program monitors and identifies the current topology and active buses where each microgrid is segmented with level order where the DG connected PCC / Common bus is of high priority (HP) level as it is the main bus connecting Quintuple structure of DC microgrid. The bus connected with DG’s and loads are in secondary level of protection. The indicated level (faulted point/location) is predicted by LOTT helps the Bidirectional Dial’s to ease the identification of faulted point and finding the shortest path from dormant bus to nearest operating DGs^24^. Bus 1 and 6 is noted as Level 1, Bus 2 and 7 as level 2, Bus 3 and load 2 as level 3, Bus 4 in level 4, Bus as level 5, and load 1 as level 6 as shown in Fig. 4 (a). The same preference is followed for all DCMG configurations and the reconfigurable grid structure with respect to LOTT algorithm is shown in Fig. 4 (b).

**Fig. 5:**
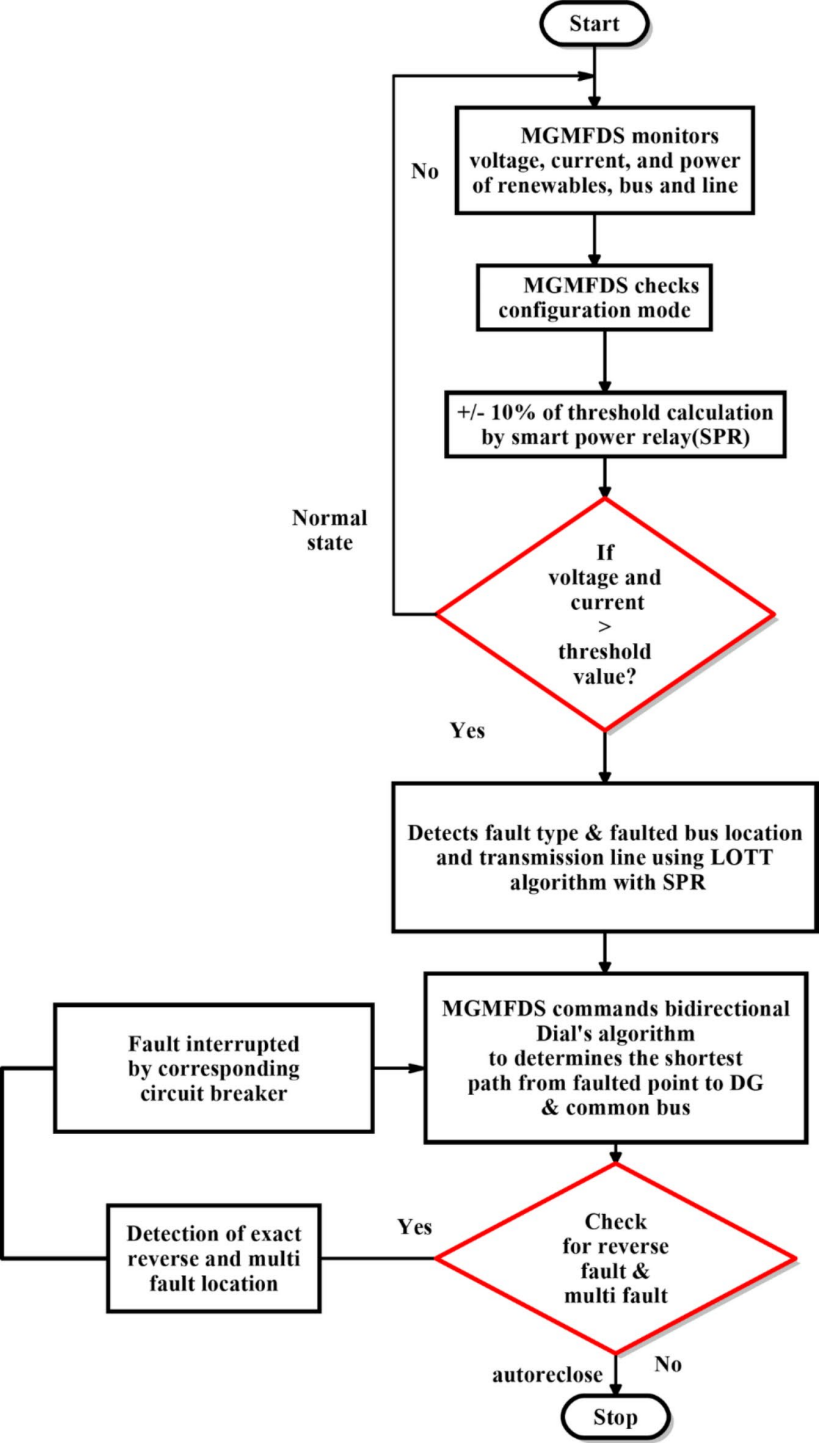
Work flow of proposed algorithm in Quintuple DC microgrid system.

The Fig. 5 shows the working flow of proposed algorithm where the MGMFDS monitors local parameters and network topology. Using a conventional overcurrent protection method, if any of these parameters exceed the predefined thresholds set by the Smart Power Relay (SPR), the LOTT algorithm determines the faulted bus and locates the fault within the system hierarchy. Based on this information, the MGMFDS employs the bidirectional Dial’s algorithm to compute the minimal distance from the fault to the nearest Distributed Generators (DGs). Subsequently, the corresponding breaker trips upon the instructions from SPR, effectively isolating the fault and thereby, the system further checks for potential reverse faults contributed due to the presence of DGs using SPR, ensuring comprehensive fault detection and interruption.

## Results and Discussions

**Fig. 6:**
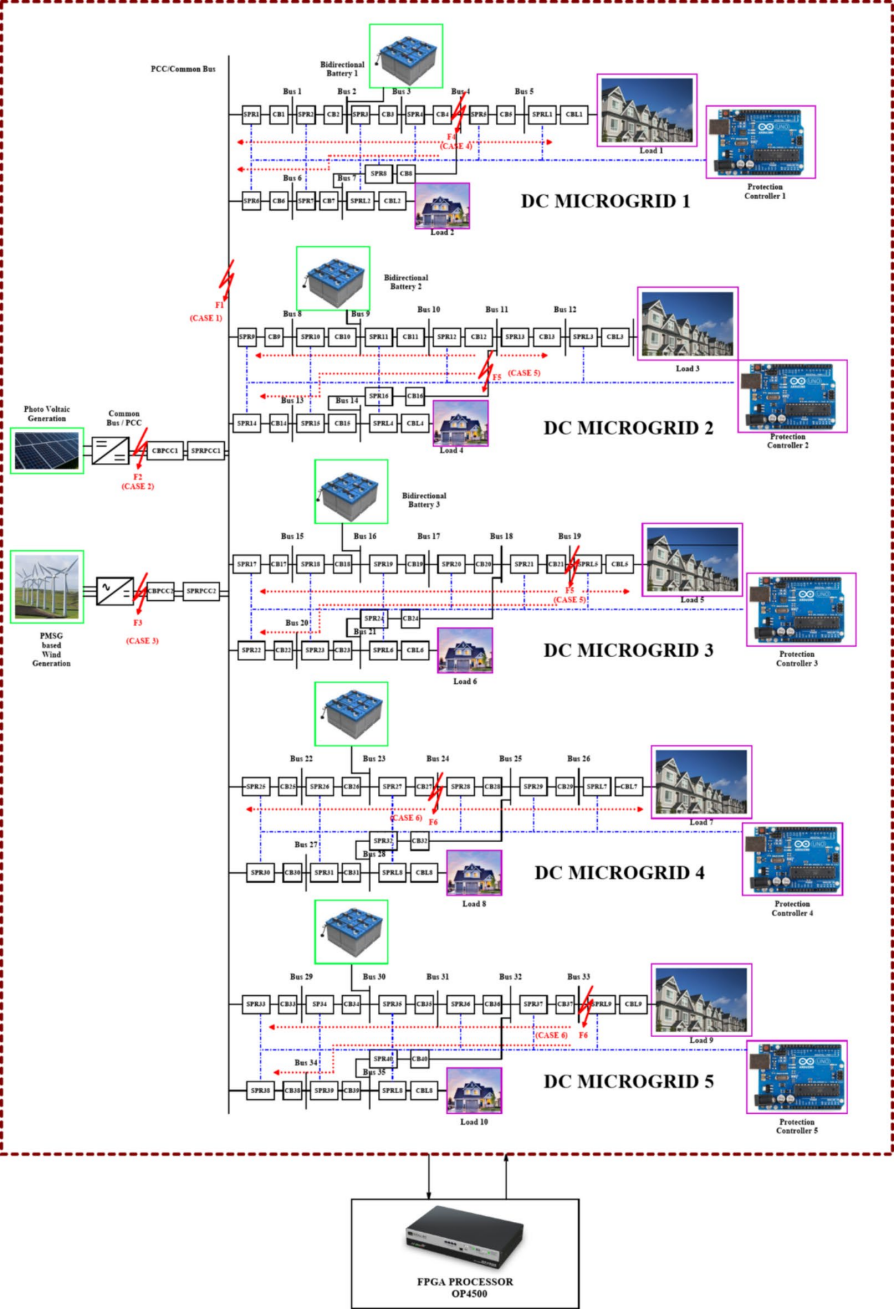
Fault analysis of 35 bus quintuple DC microgrid system.

The proposed graph algorithm with 35 bus quintuple DC microgrid system built in MATLAB-Python platform interfaced with real time simulator OP4500 and the MGMFDS. The controller with MGMFDS is fed with Python program for external verification of relays which is noted with LED in breadboard setup. The entire action of LOTT and Dial’s algorithm are tested and verified in real time simulator for Control hardware in loop testing and the efficiency is proved from various analysis carried and the results from DSO waveforms replicates its real time results of CHIL testing as shown in Fig. 6.

### Fault F1 at common bus

**Fig. 7:**
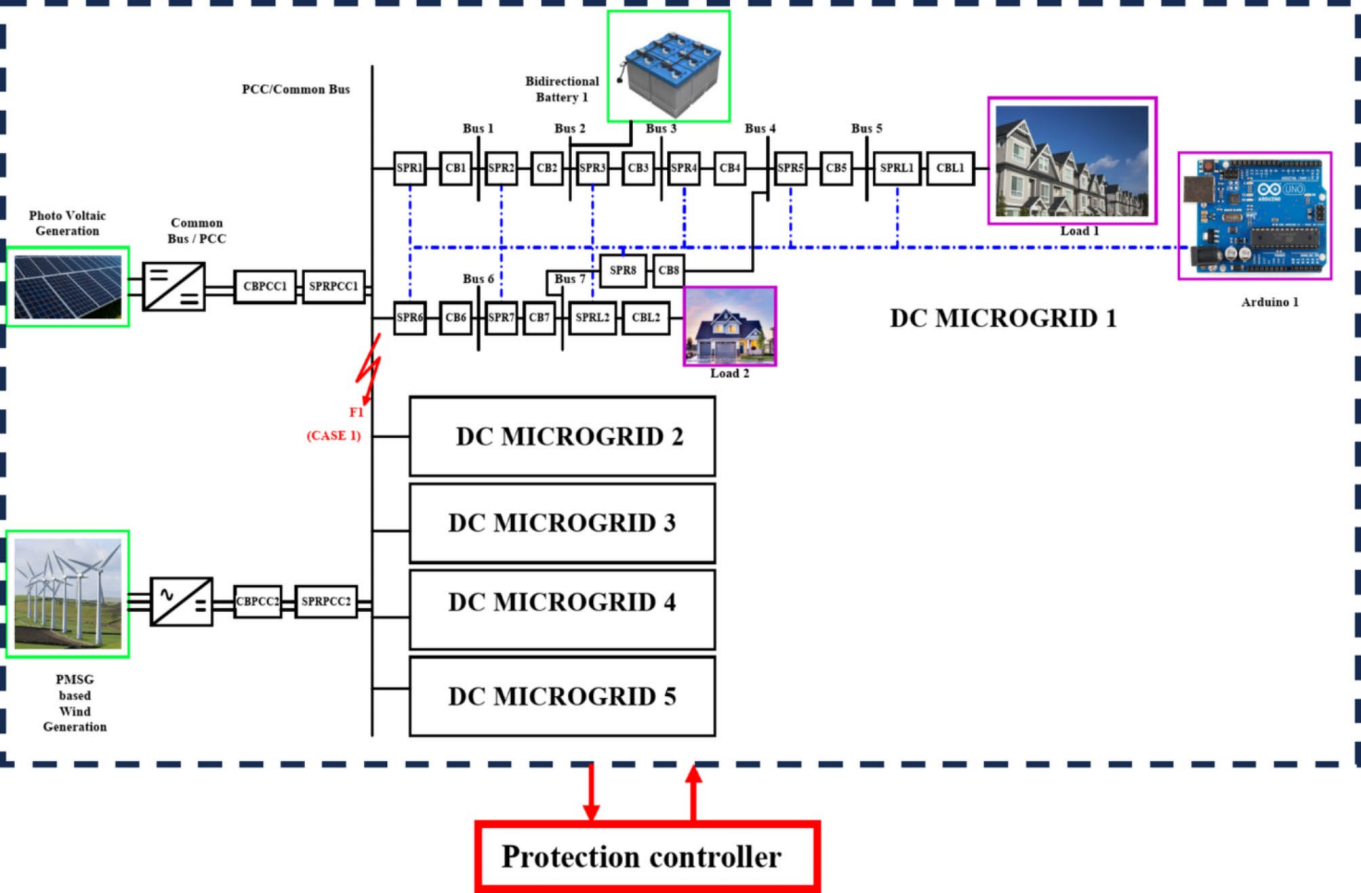
Analysing Fault F1 in common bus.

**Fig. 8:**
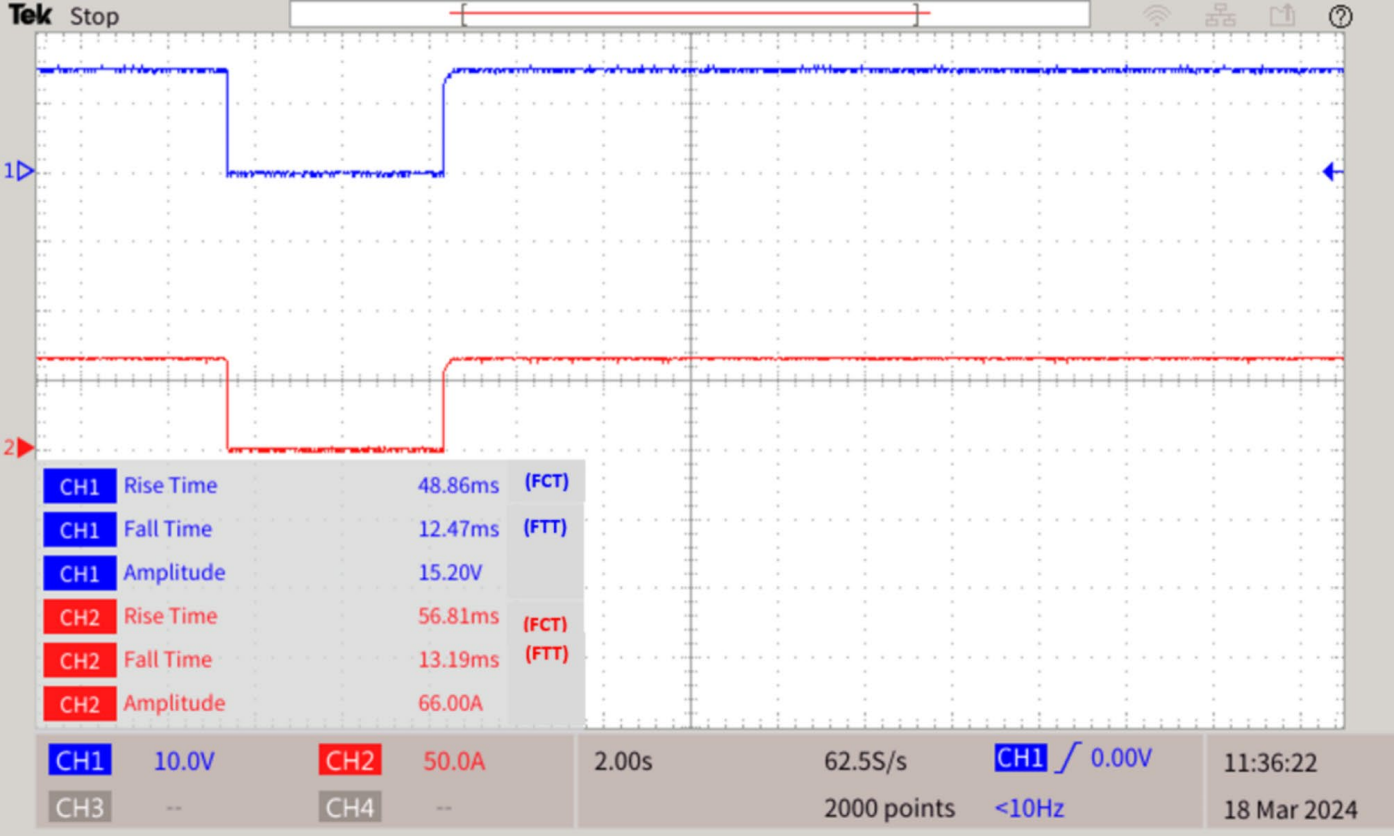
Results of a Fault F1 at common bus.

During normal operating conditions, all DCMG systems are operated at 1500 V and 66 A that provides an output power of 100 kW system. When a fault F1 occurred at common bus which is present at highest priority level, the operational buses (OB) and dormant bus (DB) are determined by the values noted by SPR monitored through MGMFDS, thereby immediately the LOTT algorithm predicts the fault location as shown in Figs. 7 and 8. The fault recognized by SPR fixes the threshold as shown in Table 2. This makes the SPRPCC 1 and 2 integrated with CBPCC 1 and 2 connected to the renewables detects and interrupts the fault, thereby the time taken to interrupt the voltage during fault i.e. fault tripping time (FTT) is 12.47ms and the time taken to reclose the breaker voltage is 48.86ms. Also, the time taken to interrupt the current during fault i.e. fault tripping time (FTT) is 13.19 ms and the time taken to reclose the breaker current is 56.81ms. The short circuit current is also interrupted by CB1,6,9,14,17,22,25,30,33,38 to ensure the safety and reliability of the DCMGs. The DSO results, as depicted in Fig. 8, assess the algorithm-driven protection showing voltage dips to zero during the bus fault, followed by CB action on the common bus.

**Table 2:** Relay threshold fixation by SPR during normal and abnormal operating conditions

Case 1	DC microgrid segment		Relay threshold fixed by SPR during fault scenarios																							
			DCMG 1				DCMG 2				DCMG 3				DCMG 4				DCMG 5				PV fed converter		Wind fed converter	
	Normal operating conditions		SPR 1		SPR 6		SPR 9		SPR 14		SPR 17		SPR 22		SPR 25		SPR 30		SPR 33		SPR 38		SPRPCC 1		SPRPCC 2	
Bus Fault at common bus F1	V (V)	I (A)	V (V)	I (A)	V(V)	I (A)	V (V)	I (A)	V (V)	I (A)	V (V)	I (A)	V(V)	I (A)	V(V)	I (A)	V(V)	I (A)	V(V)	I (A)	V(V)	I (A)	V(V)	I (A)	V (V)	I (A)
	1500	15	1349.5	21	1349.4	21	1349.2	21	1349	21	1349.1	21	1349.3	21	1349.3	21	1349.2	21	1349.6	8.6	1349.1	8.6	1348.9	40	1348	41

### Fault F2 at PV interfaced DC/DC converter

**Fig. 9:**
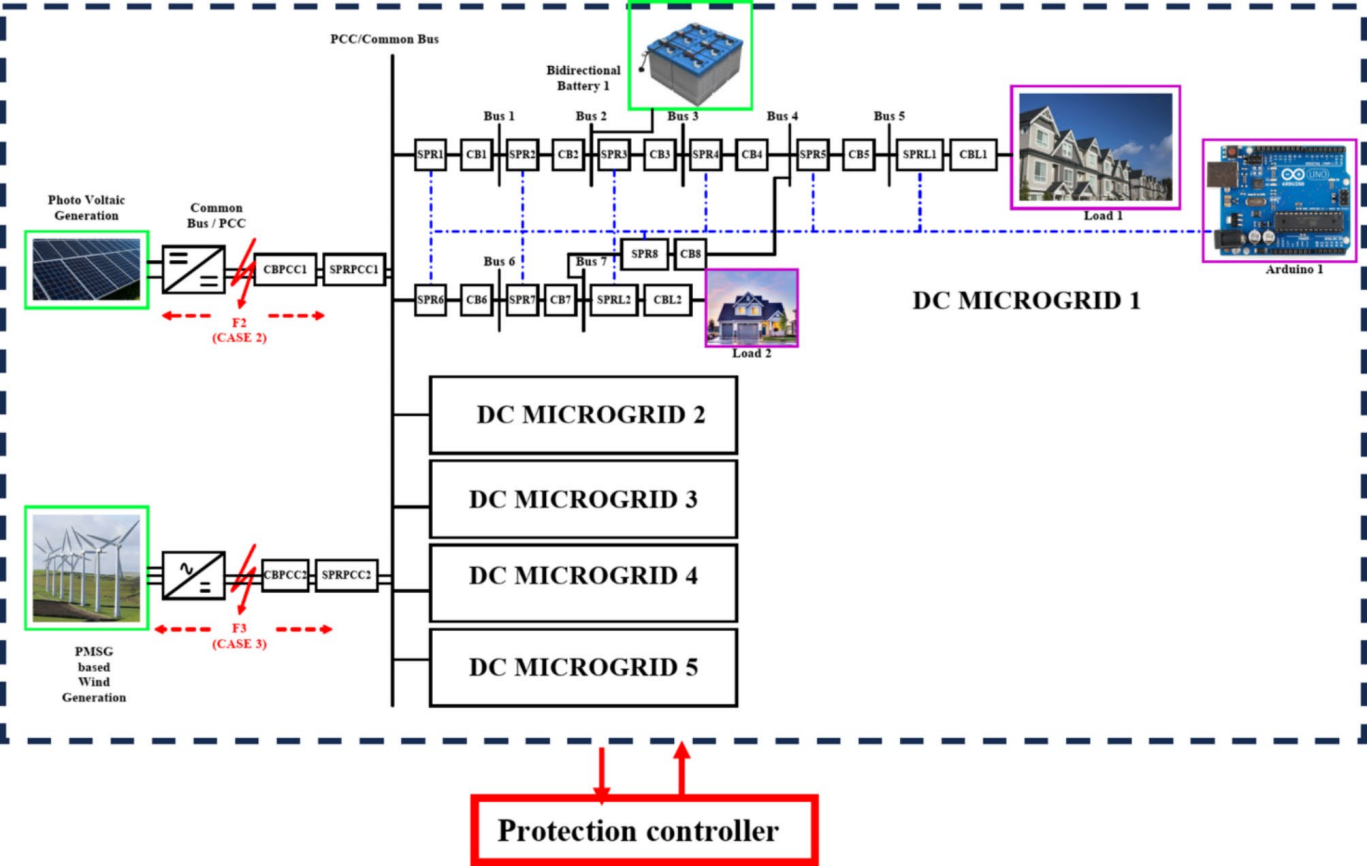
Analysing Fault F2 and F3 in 35 bus quintuple DC microgrid system.

**Fig. 10:**
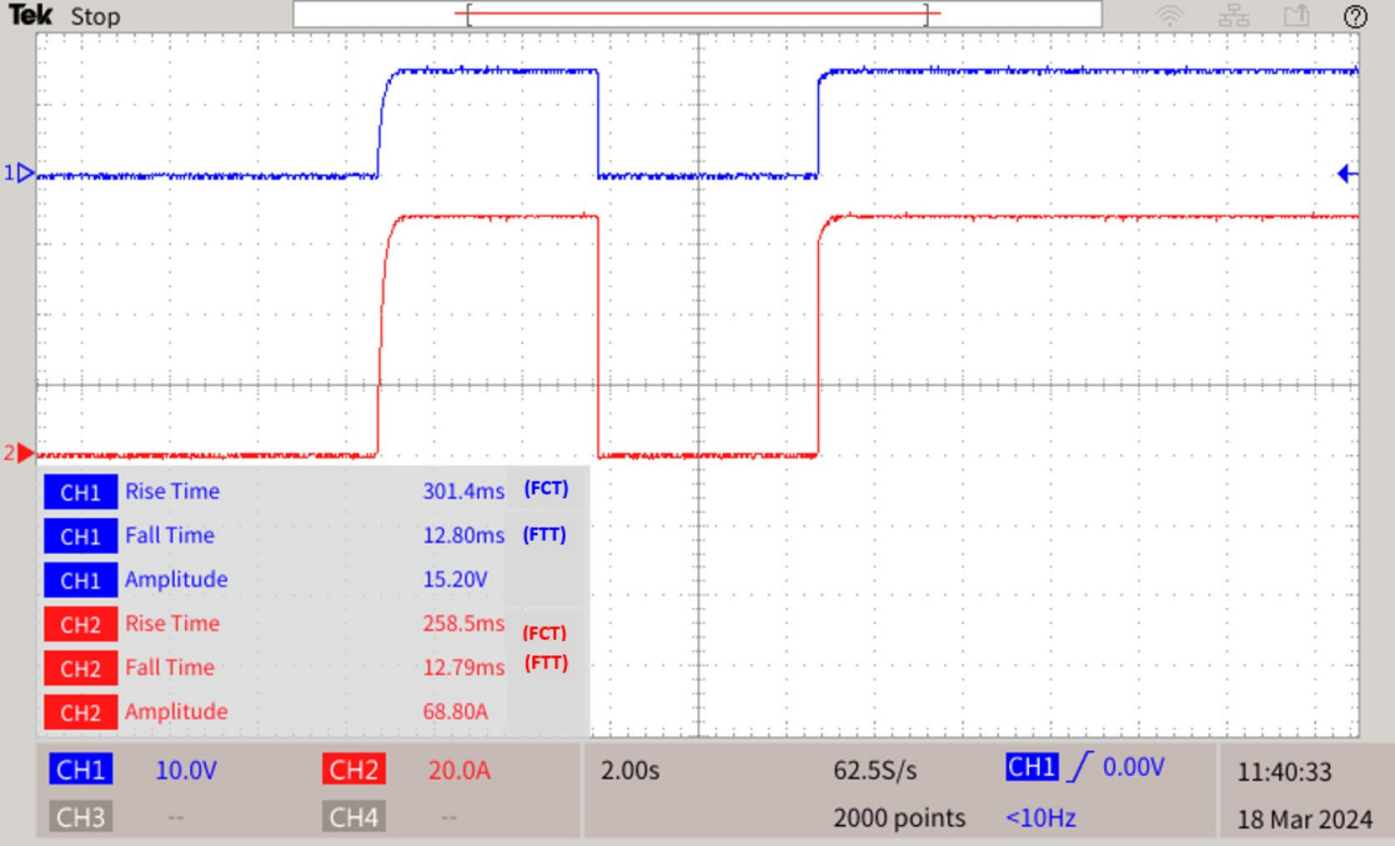
Fault F2 at PV interfaced DC/DC converter.

During a fault F2 occurred at converter connected to PV panel as shown in Fig. 9, immediately the SPRPCC 1 connected to the renewables monitored through MGMFDS detects and interrupts the fault, thereby the time taken to interrupt the voltage during fault i.e. FTT is 12.80ms and the time taken to reclose the breaker voltage i.e. FCT is 301.4ms. Also, the time taken to interrupt the current during fault i.e. FTT is 12.79 ms and the time taken to reclose the breaker current is 258.5ms as shown in Fig. 10. Therefore, the advanced graph algorithm-based protection strategy validates its effectiveness and dependability. The relay threshold fixation by SPR during normal and abnormal operating conditions are show in Table 3.

### Fault F3 at wind turbine interfaced AC/DC converter

**Fig. 11:**
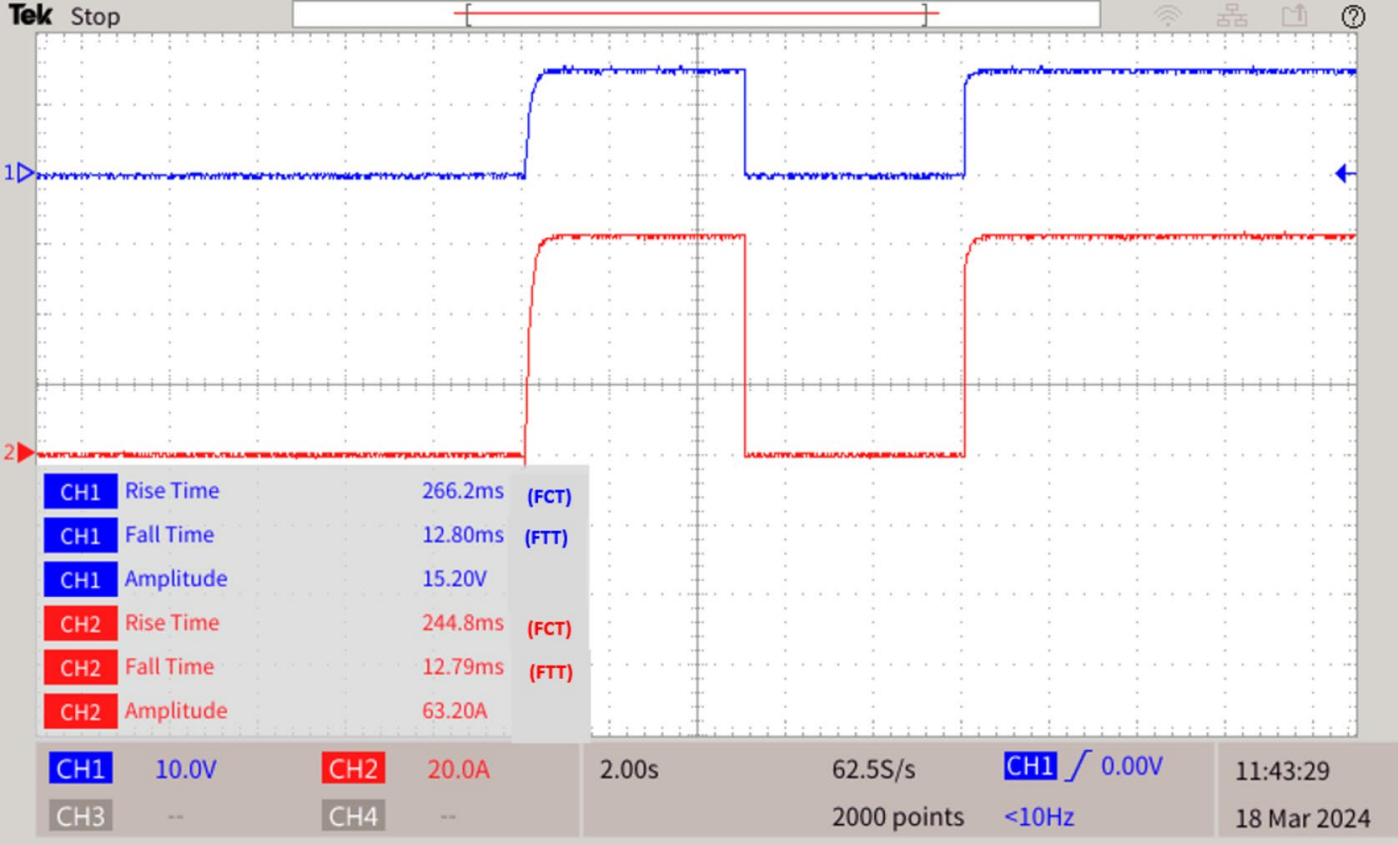
Fault F3 at wind turbine interfaced converter.

 During a fault F3 occurred at converter connected to wind turbine terminal, immediately the SPRPCC2 detects and interrupts the fault with the assistance of MGMFDS, thereby the time taken to interrupt the voltage during fault i.e. FTT is 12.80ms and the time taken to reclose the breaker voltage i.e. FCT is 266.2ms. Also, the time taken to interrupt the current during fault i.e. FTT is 12.79 ms and the time taken to reclose the breaker current is 244.8ms as shown in Fig. 11.

**Table 3:** Relay threshold fixation by SPR during normal and abnormal operating conditions.

Case 2 & Case 3	Normal operating conditions		Relay threshold fixed by SPR during fault scenarios			
			PV fed converter (Case 2)		Wind fed converter (Case 3)	
			SPRPCC 1		SPRPCC 2	
Fault F2 and F3 near converter terminals	V (V)	I (A)	V (V)	I (A)	V (V)	I (A)
	1500	15	1349.9	40.8	1349.9	36.2

### Fault F4 at bus 4

**Fig. 12:**
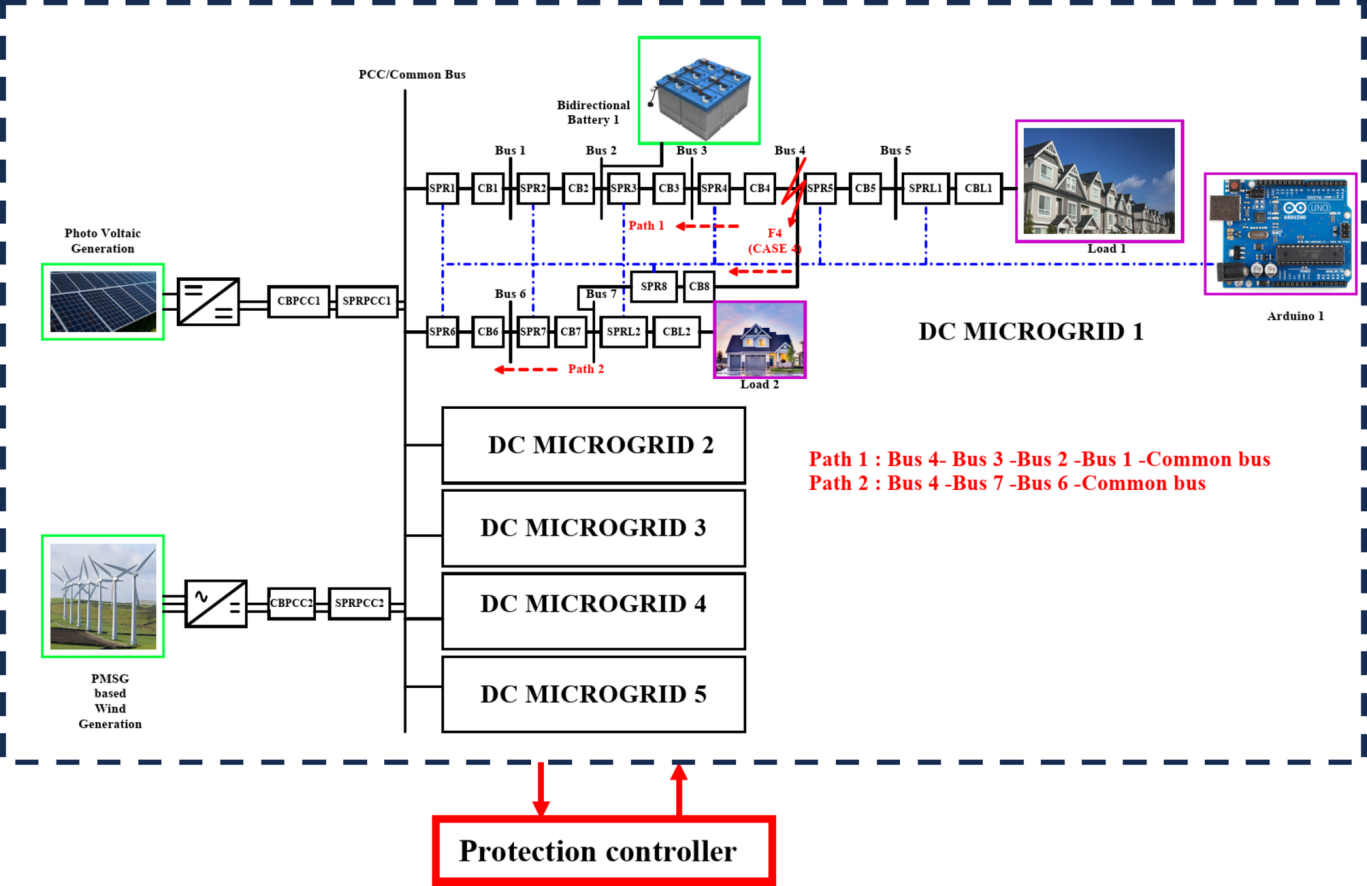
Fault F4 at bus 4.

**Fig. 13:**
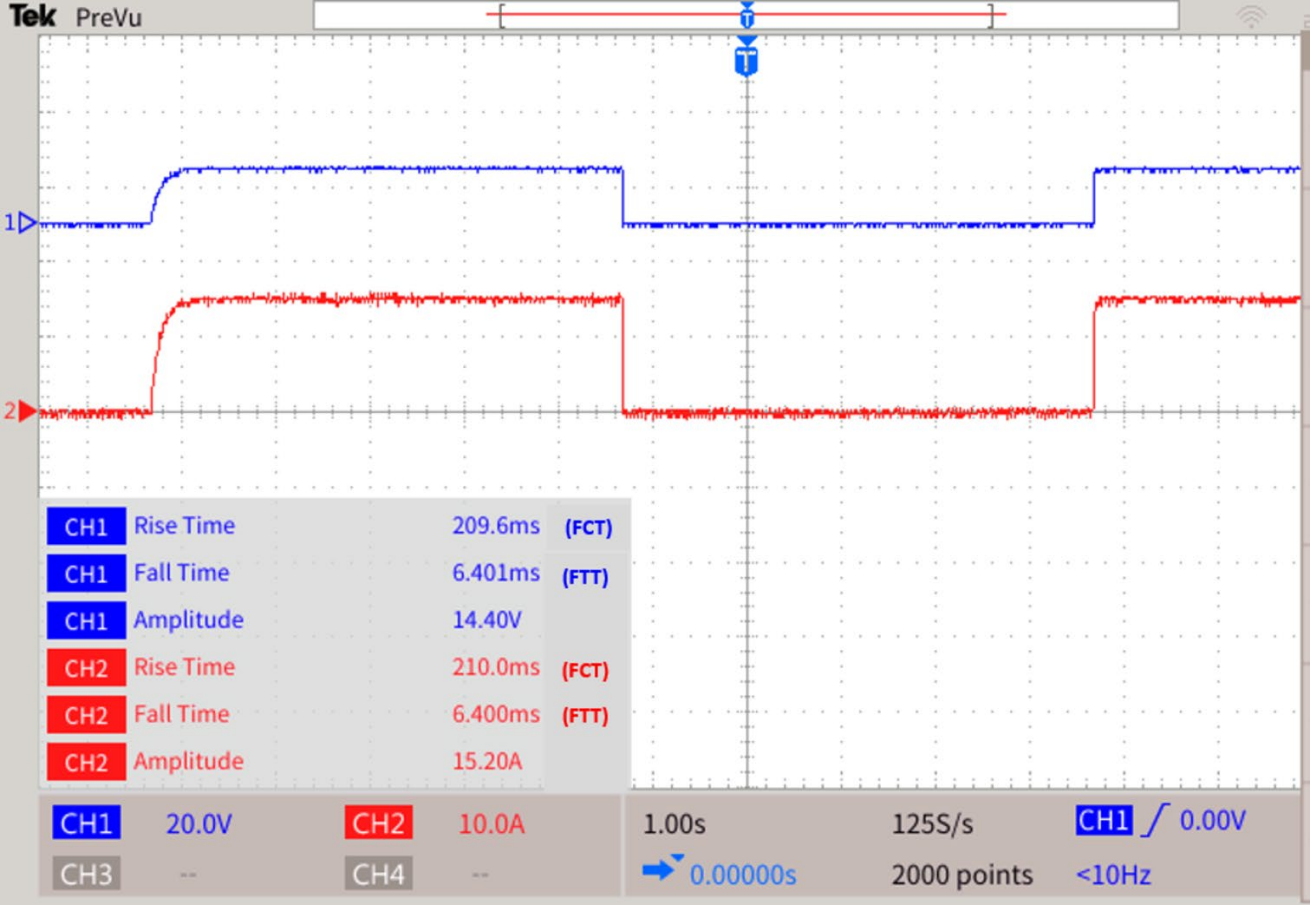
Fault F4 at bus 4.

 Bus 4 which maintains a voltage of 1500 V, and an operating current of 15.2 A experiences a fault F4. The LOTT system with the assistance of SPR predicts the presence of fault with maximum efficacy and further the bidirectional dial’s estimate the shortest possible path from the disrupted section to the most adjacent functional distributed power generation. As shown in Fig. 12, the fault path is Path 1: Bus 3 − 2–1 - Common Bus and Path 2: Bus 7 − 6 -Common bus. On comparison, the path Bus 3 − 2–1 – Common bus is prioritized first as the existence of bidirectional battery in the relevant path. Though the most effective and streamlined path has been identified as the nodes traversing through Bus 7 − 6 - Common Bus, the DG connected bus is prioritized first. As the preference logic enabled in MGMFDS with respect to DG connection, the CBs are tripped in the order CB4, CB5 and CB8. With the proposed scheme, the CB4, CB5 and CB8 voltage is tripped off taking a total transit time i.e. FTT for CB4 is estimated to be 6.401ms and the auto reclosing interval i.e. FCT of 209.6ms. Also, the CB4 current is tripped off taking a total transit time i.e. FTT estimated to be 6.400ms and the auto reclosing interval i.e. FCT of 210ms as shown in Fig. 13. The relay threshold fixation by SPR during normal and abnormal operating conditions.

For fault F4 are given in Table 4.

**Table 4:** Relay threshold fixation by SPR during normal and abnormal operating conditions

**Case 4**	**Normal operating conditions**		**Relay threshold fixed by SPR during fault scenarios**					
			**DCMG 1**					
			**SPR 4**		**SPR 5**		**SPR 8**	
Fault at Bus 4	V (V)	I (A)	V (V)	I (A)	V (V)	I (A)	V (V)	I (A)
	1500	15	1349.8	21.8	1349.8	21.6	1349.9	21.5

### Simultaneous fault (SF) F5 at TL11-14 and Bus 19 at DC microgrid 2 and 3

**Fig. 14:**
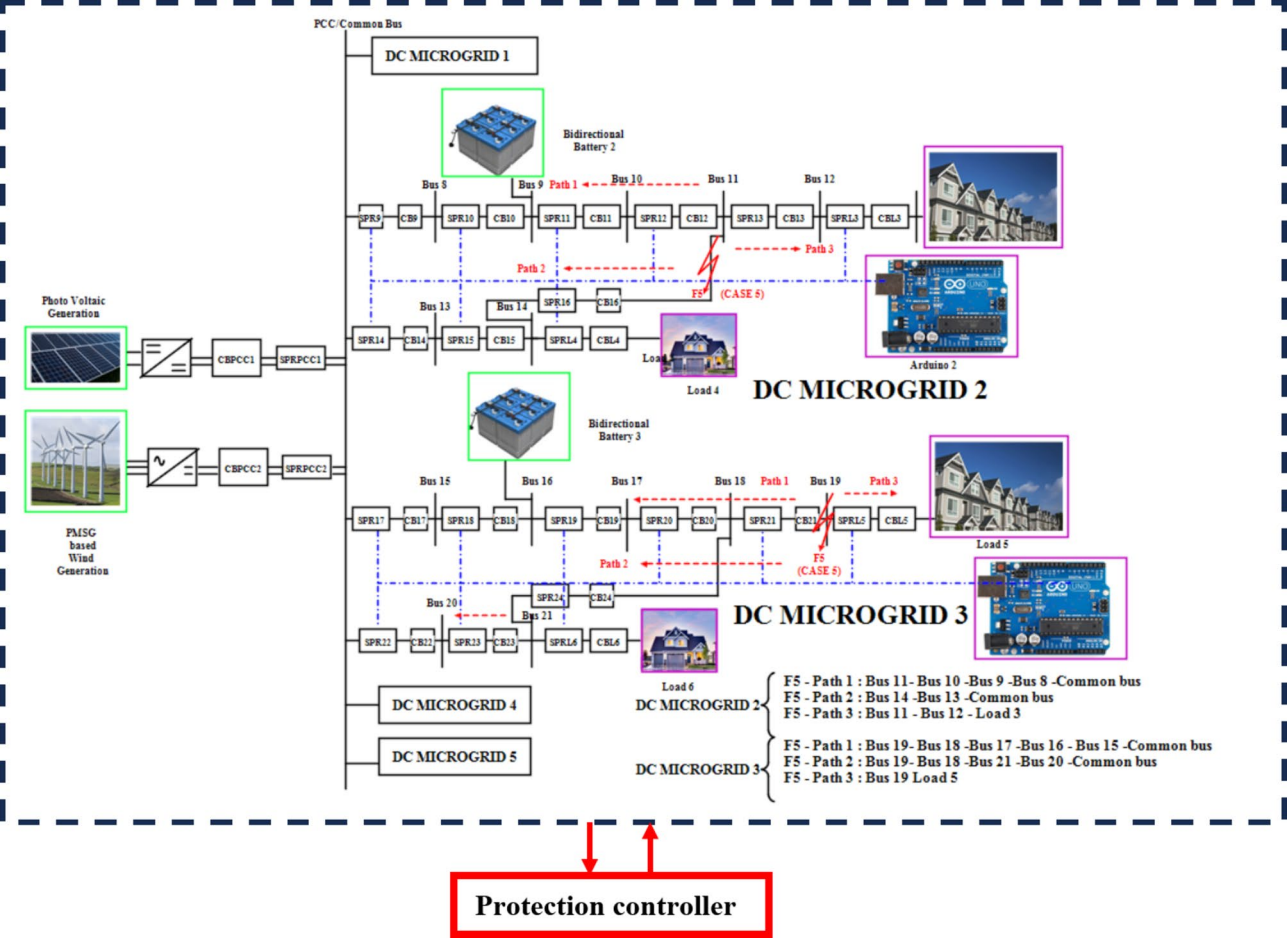
Pictorial representation of Simultaneous fault (SF) F5 at TL11-14 and Bus 19 at DC microgrid 2 and 3.

**Fig. 15:**
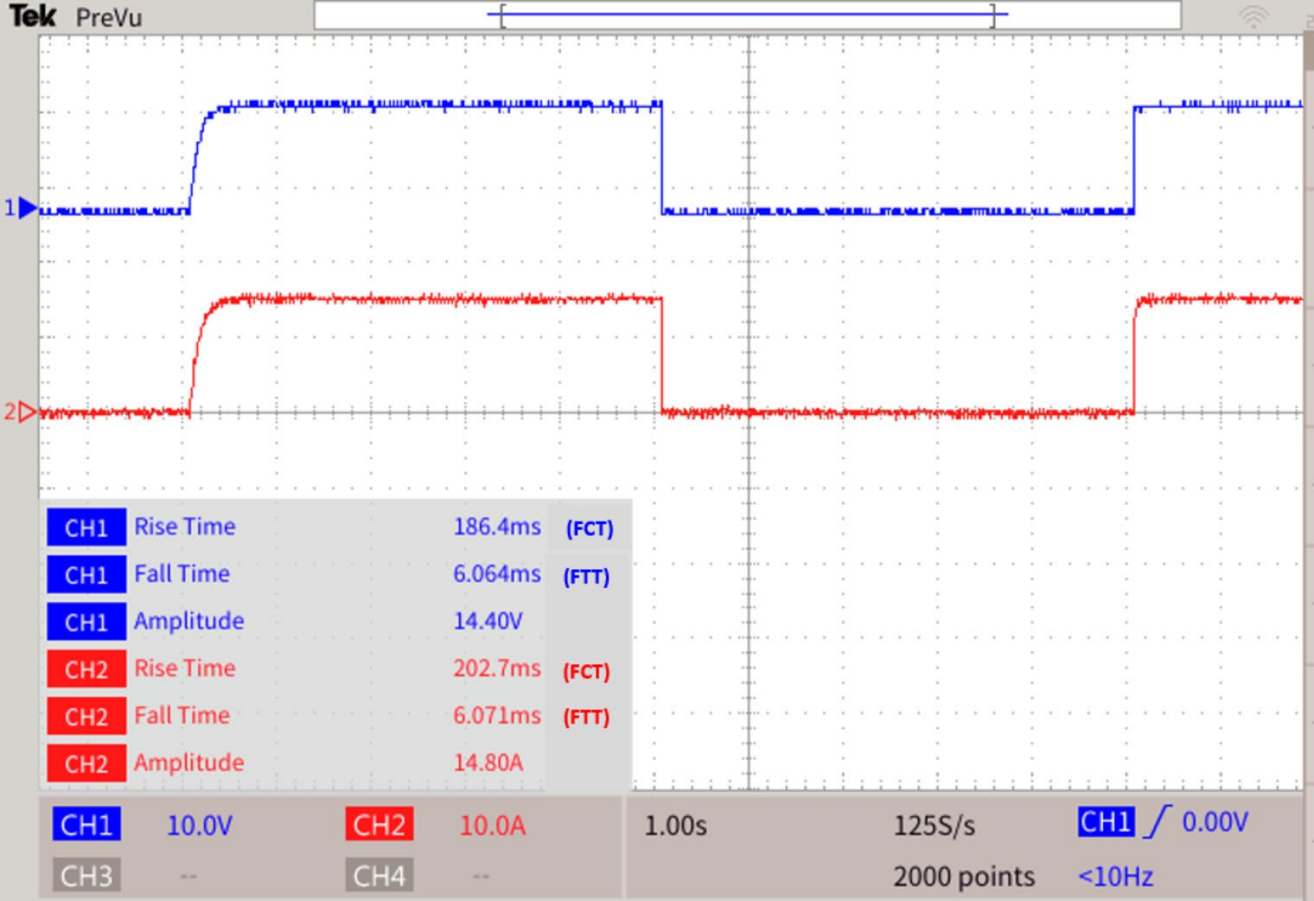
Simultaneous fault (SF) F5 at TL11-14 and Bus 19 at DC microgrid 2 and 3.

SF F5 occurs at TL11 −14 (LL fault) and Bus 19, where the LOTT system with the assistance of MGMFDS integrated with SPR predicts the presence of fault (Table 5) with maximum efficacy and further the bidirectional dial’s estimate the shortest possible path from the disrupted section to the most adjacent functional distributed power generation. As shown in Fig. 14, the fault path is Path 1: Bus 11–12 – Load 3, Path 2: Bus 14 – Load 4, Path 3: Bus 11 − 10–9 − 8 -Common bus and Path 4: Bus 14–13 – Common bus. The most minimal distance path has been identified as Path 2: Bus 14 – Load 4 with a weight of 4 as the distance taken between each bus as 2. With the proposed scheme, the CB16 and CBL4 voltage is tripped off. The total transit time for CB 16 interruption is i.e. FTT estimated to be 6.401ms and the auto reclosing interval i.e. FCT of 209.6ms. Also, the current is tripped off by CB16 takes a total transit time i.e. FTT estimated to be 6.400ms and the auto reclosing interval i.e. FCT of 210ms. as shown in Fig. 15. Simultaneously Bus 19 is affected by fault resulting in tripping the circuit breakers CBL5 and CB21. The proposed algorithm proves its uniqueness in clearing the fault for simultaneous fault conditions.

**Table 5:** Relay threshold fixation by SPR during normal and abnormal operating conditions

**Case 5**	**Normal operating conditions**		**Relay threshold fixed by SPR during fault scenarios**									
			**DCMG 2**						**DCMG 3**			
			**SPR 12**		**SPR 13**		**SPR 16**		**SPR 21**		**SPRL5**	
Simultaneous fault (SF) F5 at TL11-14 and Bus 19 at DC microgrid 2 and 3	V (V)	I (A)	V (V)	I (A)	V (V)	I (A)	V (V)	I (A)	V (V)	I (A)	V (V)	I (A)
	1500	15	1349.2	21.1	1349.4	21.3	1349.4	21.1	1349.9	21.6	1349.8	21.8

### Algorithm Operations during the disconnection of two DCMGs and fault F6 at Bus 24 and TL 33 - load 9 as multi fault scenario

**Fig. 16:**
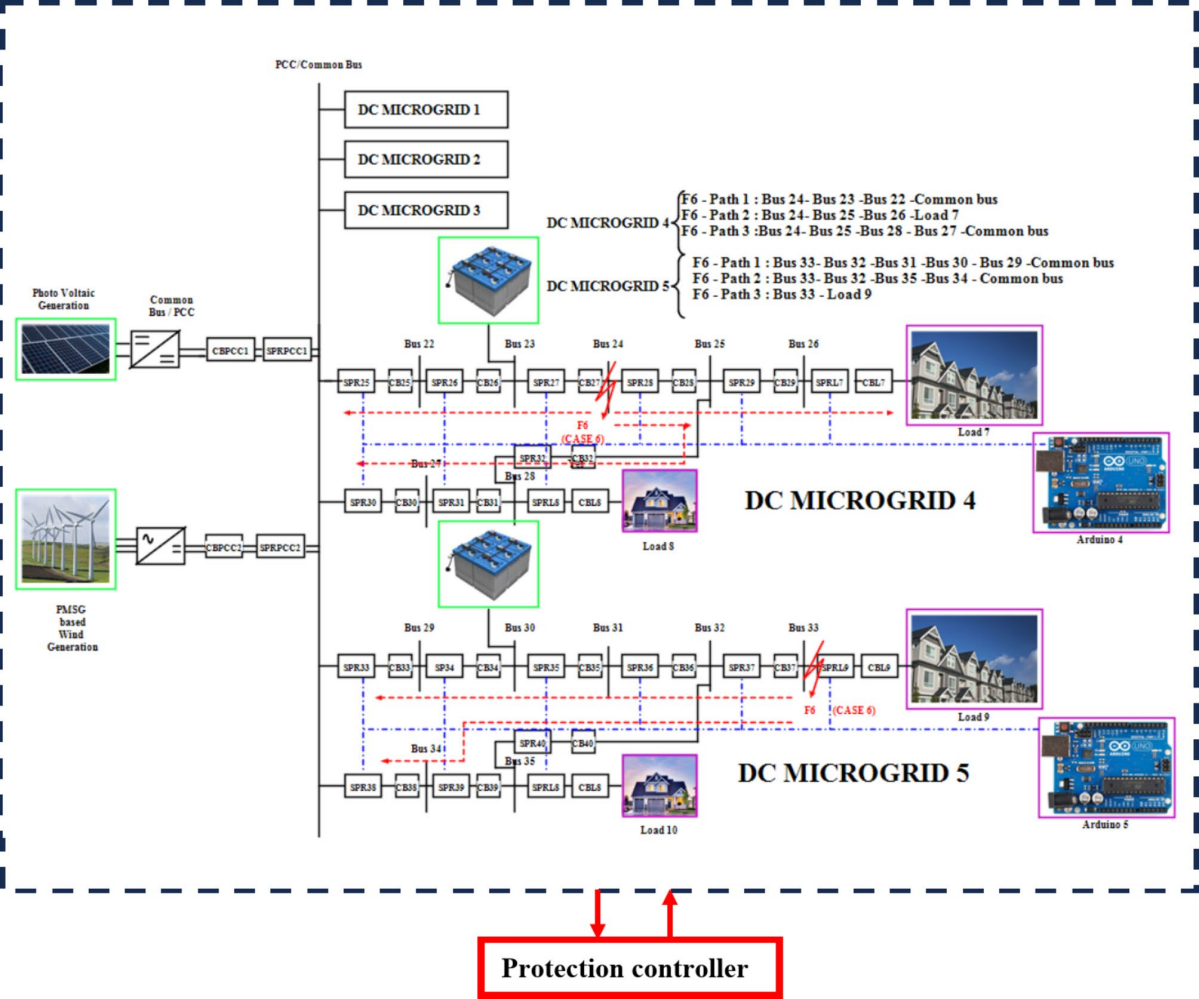
During the disconnection of two DCMGs and fault F6 at Bus 24 and TL 33 - load 9 as (MF) scenario.

**Fig. 17:**
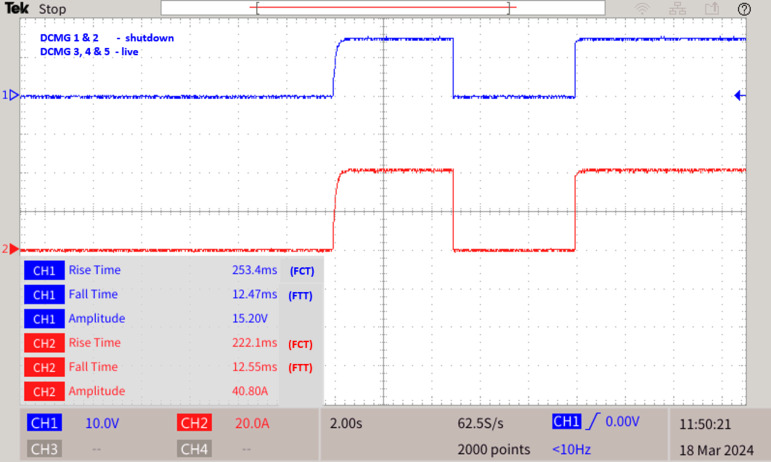
During the disconnection of two DCMGs and fault F6 at Bus 24 and TL 33 - load 9 as (MF) scenario.

As shown in Fig. 16, when DCMG 1 and 2 shutdowns and DCMG 3,4 and 5 is in operating state, if a fault F6 occurs at Bus 24 and LG fault in transmission line TL 33-load 9, immediately the LOTT system with the assistance of SPR present with MGMFDS predicts the presence of fault F6 with maximum efficacy and further the bidirectional dial’s estimate the shortest possible path from the disrupted section to the most adjacent functional distributed power generation. The fault path for TL 33 - load 9 is Path 1: Bus 33 − 32–31 – 30–29 - common bus, Path 2: Bus 32–35 −34- common bus, Path 3: Bus 33 -Load 9,and Path 4: Bus 32–35 - Load 10. On comparison, the least distance path with priority factor consideration by MGMFDS the path 3 has been identified as minimal distance path. With the proposed scheme, the CBL9 and CB37 is tripped where the CL9 takes a total transit time i.e. FTT estimated to be 6.401ms and the auto reclosing interval i.e. FCT of 209.6ms. Also, the CBL9 fault current is tripped off taking a total transit time i.e. FTT estimated to be 6.400ms and the auto reclosing interval i.e. FCT of 210ms. as shown in Fig. 17. Simultaneously Bus 24 is affected by fault resulting in tripping the circuit breakers CB27 and CB28. The relay threshold fixation by SPR during normal and abnormal operating conditions are show in Table 6.

The Relay threshold fixed by SPR during normal and abnormal operating conditions for quintuple DC microgrid structure is shown in Tables 7, 8 and 9 and the highlighted values noted are threshold fixed for various fault occurs at different locations. With respect to these threshold values, the LOTT algorithm and bidirectional dial’s algorithm determines the exact fault location and fault path with minimal distance resulting in detecting and interrupting the fault.

**Fig. 18:**
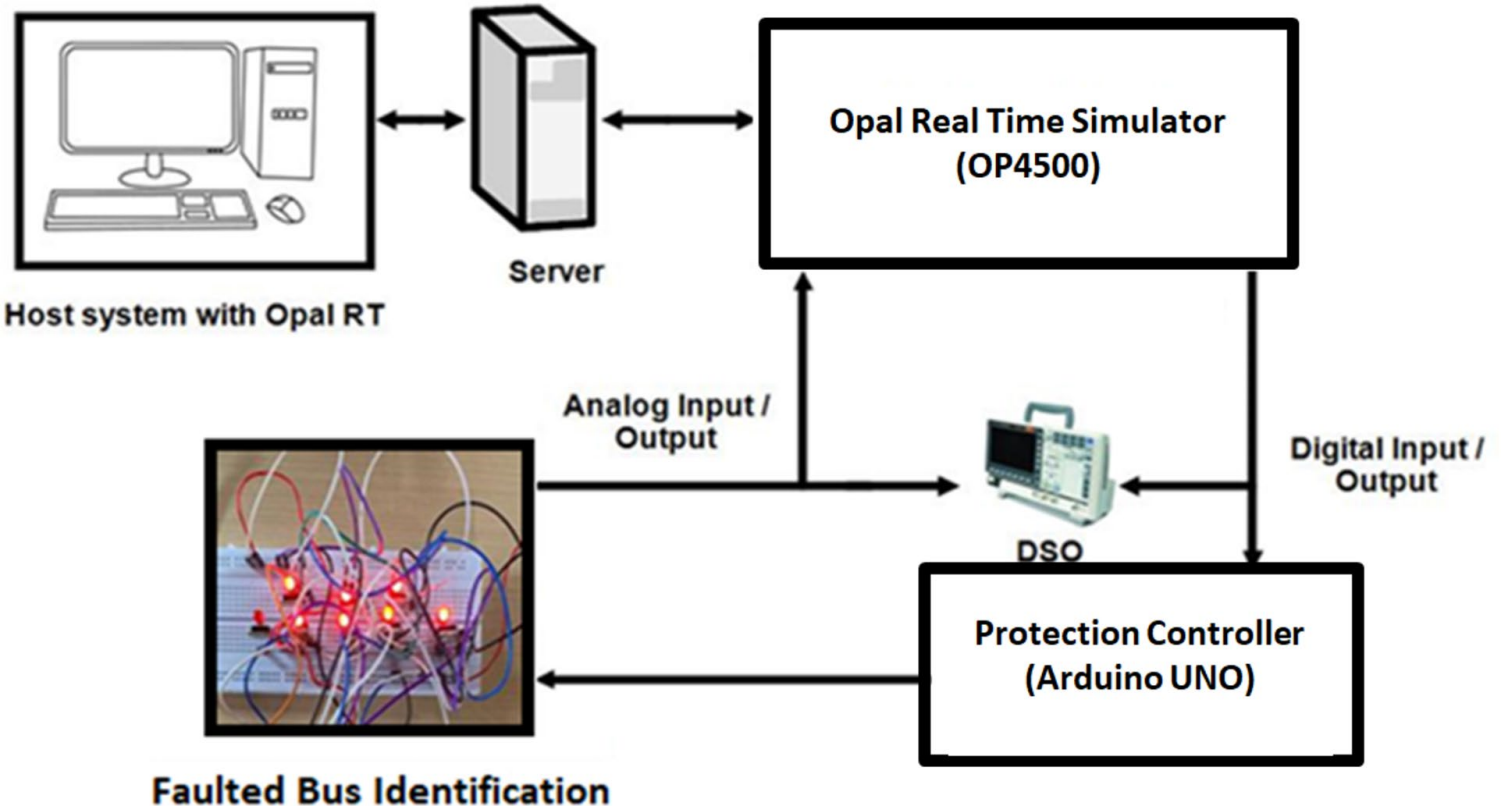
Experimental setup of quintuple DC microgrid system.

This study is evaluated through model-in-the-loop (MIL) and control hardware-in-the-loop (CHIL) testing, which offers significant advantages by enabling thorough examination of relay communication using an Arduino UNO board as illustrated in Fig. 18. This setup minimizes the need for external data transmission to display relay conditions, thus ensuring signal coherence. This section details the testing of a DC microgrid using a OP4500 real-time simulator (RTS) within the RT-Lab environment and the simulink models of a Quintuple DC system, incorporating photovoltaic (PV) panels, wind energy conversion systems (WECS), batteries, and loads, are interfaced with Opcomm and Opctrl RT controller blocks. These blocks are connected to the OP4500 simulation target via a Transmission Control Protocol (TCP) / Internet Protocol (IP) communication network on the host computer. The voltage levels of each microgrid are set at 1500 V and 15 A, constrained to a range of ± 16 V to meet real-time simulator compatibility requirements and a voltage gain is applied to facilitate these adjustments.

**Table 6:** Relay threshold fixation by SPR during normal and abnormal operating conditions

**Case 6**	**Normal operating conditions **		**Relay threshold fixed by SPR during fault scenarios**							
			**DCMG 4**				**DCMG 5**			
			**SPR 27**		**SPR 28**		**SPR 37**		**SPRL9**	
During the disconnection of two DCMGs and fault F6 at Bus 24 and TL 33 - load 9 as (MF) scenario	V (V)	I (A)	V (V)	I (A)	V (V)	I (A)	V (V)	I (A)	V (V)	I (A)
	1500	15, 6	1349.8	21.4	1349.9	21.4	1349	8.8	1348.9	8.6

The Tables 7, 8 and 9 shows the faulted impression thereby fixes the relay threshold through SPR for DC microgrid 1,2,3,4 and 5. For fault F1, SPR1 determines and fixes a threshold of 1349.5 V and 21.1 A. As the fault is at common bus, all the SPR connected to it (SPR6,9,14,17,22,25,30,33,38) fixes the threshold and the algorithm identifies the exact fault location. Therefore, the above-mentioned SPR acts for the fault F1. For fault F2 and F3, the SPRPCC1and SPRPCC2 fixes fault threshold value of 1349.9 V and 40.8 A and 1349.9 and 36.2 A respectively. For fault F4, SPR 4, 5 and 8 acts whereas for fault F5 SPR 12, 13,16,21 and SPRL5 fixes the threshold, thereby the algorithm works in determining the OB and DB. For fault F6, SPR 27,28,37 and SPRL9 acts during the disconnection of two microgrid.

**Table 7:** Relay threshold fixed by SPR during normal and abnormal operating conditions for DC microgrid 1 and 2

DC microgrid segment	Operating conditions / Relay points	Normal operating conditions		Abnormal operating conditions											
				Relay threshold fixed by SPR during fault scenarios											
				F1		F2		F3		F4		F5		F6	
		Voltage (V)	Current (A)	Voltage (V)	Current (A)	Voltage (V)	Current (A)	Voltage (V)	Current (A)	Voltage (V)	Current (A)	Voltage (V)	Current (A)	Voltage (V)	Current (A)
DC microgrid 1	SPR1	1500	15	1349.5	21.1	1496	15	1499	15.2	1495	15.4	1501	14.9	1499	15.3
	SPR2	1506	15	1501	15.2	1501	15.2	1498	15.3	1496	14.2	1502	15.1	1490	15
	SPR3	1505	15.2	1502	14.9	1508	15	1496	15.8	1501	15.6	1501	15	1488	15
	SPR4	1499	15	1506	15.2	1508	15.3	1501	15	1349.8	21.8	1508	15.2	1489	15.2
	SPR5	1498	15.4	1507	15.3	1507	15	1499	15.1	1349.9	21.6	1508	14	1508	14.8
	SPR6	1496	15	1349.4	21.2	1505	15.4	1501	15.3	1499	15.6	1508	14.2	1499	14.2
	SPR7	1501	15	1505	15.1	1500	14.2	1495	15	1491	14.9	1508	14.3	1500	14
	SPR8	1508	15	1501	15.2	1501	15.6	1496	15	1349.9	21.5	1507	15.2	1499	14.2
	SPRL1	1508	15.2	1499	15.1	1499	14.9	1501	15.2	1501	14.2	1505	15.6	1489	14.3
	SPRL2	1507	15	1501	15	1501	15	1502	15	1495	15.6	1502	15.8	1507	15.2
DC microgrid 2	SPR9	1505	15.3	1349.2	21.1	1496	15.1	1501	15.4	1499	14.9	1500	15.9	1506	14.8
	SPR10	1502	15	1498	14.8	1501	15.3	1508	14.2	1491	15	1501	15.6	1507	14.2
	SPR11	1503	15.2	1496	14.9	1508	15	1508	15.6	1499	15.1	1500	15.6	1506	14.2
	SPR12	1500	14.9	1501	14.7	1508	15.2	1499	14.9	1498	15.2	1349.2	21.1	1502	14.3
	SPR13	1501	15.1	1502	14.9	1507	15	1500	15.1	1496	15.1	1349.4	21.3	1501	14.8
	SPR14	1499	15	1349	21.3	1505	15.2	1499	15	1501	15.2	1501	15.2	1501	14.8
	SPR15	1501	15.4	1507	15.4	1502	14.8	1489	15.2	1501	15	1500	15.1	1495	14.8
	SPR16	1495	15.2	1488	15	1503	14.2	1507	15.2	1499	15.1	1349.4	21.1	1494	14.9
	SPRL3	1499	15.2	1506	15	1498	14	1505	15	1489	14.8	1495	15	1499	15
	SPRL4	1491	15.4	1501	15	1495	14.1	1500	15.1	1507	14.9	1499	15.1	1500	15.1

**Table 8:** Relay threshold fixed by SPR during normal and abnormal operating conditions for DC microgrid 3 and 4

DC microgrid segment	Operating conditions / Relay points	Normal operating conditions		Abnormal operating conditions											
				Relay threshold fixed by SPR during fault scenarios											
				F1		F2		F3		F4		F5		F6	
		Voltage (V)	Current (A)	Voltage (V)	Current (A)	Voltage (V)	Current (A)	Voltage (V)	Current (A)	Voltage (V)	Current (A)	Voltage (V)	Current (A)	Voltage (V)	Current (A)
DC microgrid 3	SPR17	1499	14.2	1349.1	21	1488	15	1501	15.3	1505	15	1491	15.2	1501	15.2
	SPR18	1498	15.6	1499	15.2	1481	15.1	1499	15.4	1500	15.1	1499	15.2	1502	15
	SPR19	1496	14.9	1496	15.1	1489	15.6	1499	15.2	1501	15.2	1498	15.3	1501	15.3
	SPR20	1501	15	1499	15	1486	15.8	1501	15	1495	15.2	1499	15	1501	15
	SPR21	1502	15.1	1500	15.6	1499	15.9	1495	15.3	1499	14.8	1349.9	21.6	1495	15.2
	SPR22	1503	15.3	1349.3	21.2	1500	15.6	1501	15	1481	14.8	1500	15.1	1496	15
	SPR23	1504	15.4	1499	15.6	1499	15.8	1502	15.2	1483	14.8	1501	15.2	1497	15.2
	SPR24	1509	15.5	1496	14.9	1489	15.2	1503	14.9	1500	14.9	1500	14.9	1497	15.1
	SPRL5	1507	15	1500	15	1488	15	1502	15.1	1501	14.7	1349.8	21.8	1498	15
	SPRL6	1488	15.1	1501	15.1	1487	15.1	1502	14.6	1502	14.6	1499	15.2	1491	15.1
DC microgrid 4	SPR25	1506	15	1349.3	21.1	1480	15.3	1502	14.8	1501	14.6	1498	14.9	1490	14.9
	SPR26	1501	15.1	1522	15.1	1490	15.4	1500	15.2	1501	15	1495	15.1	1491	14.8
	SPR27	1499	15.2	1518	15	1498	15.5	1500	15.1	1500	15.2	1498	15.2	1349.8	21.4
	SPR28	1501	14.9	1517	15.2	1497	15	1501	15	1499	15	1490	14.9	1349.9	21.4
	SPR29	1495	14.8	1511	14.8	1496	15.1	1502	15.1	1498	15.1	1488	14.8	1500	14.8
	SPR30	1500	15.1	1349.2	21.1	1499	15.9	1499	14	1495	15.2	1489	14.8	1500	15.2
	SPR31	1496	15	1501	15.2	1507	15.6	1498	15	1495	14.9	1501	14.8	1501	15.1
	SPR32	1508	15.2	1495	14.9	1488	15.8	1497	15.1	1484	15.2	1502	14.9	1502	15
	SPRL7	1511	14.8	1500	14.8	1506	14.9	1499	15.3	1482	15.1	1501	15	1499	15.1
	SPRL8	1515	15.1	1496	15.1	1501	14.9	1491	15.4	1480	15	1501	15.1	1498	14.8

**Table 9:** Relay threshold fixed by SPR during normal and abnormal operating conditions for DC microgrid 5 and SPR interfaced with renewables.

DC microgrid segment	Operating conditions / Relay points	Normal operating conditions		Abnormal operating conditions											
				Relay threshold fixed by SPR during fault scenarios											
				F1		F2		F3		F4		F5		F6	
		Voltage (V)	Current (A)	Voltage (V)	Current (A)	Voltage (V)	Current (A)	Voltage (V)	Current (A)	Voltage (V)	Current (A)	Voltage (V)	Current (A)	Voltage (V)	Current (A)
**DC microgrid 5**	**SPR33**	1508	6.1	**1349**	**8.6**	1499	6	1492	6.2	1481	6.1	1495	6.1	1499	6
	**SPR34**	1509	6.6	1509	6.6	1499	6.2	1493	6	1483	6.2	1500	6.2	1497	6.1
	**SPR35**	1504	6	1507	6	1489	6.1	1495	6.1	1485	5.9	1501	6.3	1490	6.2
	**SPR36**	1499	6.8	1488	6.8	1488	6.1	1496	6.2	1488	6	1502	6.2	1489	6.1
	**SPR37**	1499	6	1506	6.6	1502	6.1	1499	6.3	1490	6.2	1501	6	**1349**	**8.8**
	**SPR38**	1496	6	**1349.1**	**8.6**	1503	6.3	1496	6	1488	6.3	1500	6	1488	6.6
	**SPR39**	1500	6.1	1499	6	1504	6.2	1496	6	1489	6	1499	6.2	1489	6.6
	**SPR40**	1508	6.1	1499	6.2	1509	5.9	1499	6	1487	6.2	1498	6.3	1489	6.5
	**SPRL9**	1488	6.9	1496	6.3	1508	5.8	1498	6.8	1486	6.3	1501	6.2	**1348.9**	**8.6**
	**SPRL10**	1489	7	1500	6.5	1511	6	1498	6.6	1489	6.6	1502	6.6	1491	6.1
**PV interfaced DC - DC converter**	**SPRPCC1**	1500	33	1503	32.9	**1349.9**	**40.8**	1497	32.6	1504	33.2	1501	32.8	1499	33
**Wind turbine interfaced AC - DC converter**	**SPRPCC2**	1501	29	1513	29.8	1503	29.9	**1349.9**	**36.2**	1506	29.1	1502	28.9	1501	29.3

### Comparitive analysis of various aspects of existing protection methodologies

Table 10analyses some of the existing benchmark systems with various parametric features such as rapidity of fault response, adaptive feature, and communication interface with result validation. The performance of all the communication-based protection methods serves to be ideal than the other methods, except limitations such as restricted network topology and unavailable support for multi-DC microgrid. In [11&12], the rapid response, adaptive nature in grid protection, communication interface is same but the protection strategies are not tested in multi-DC topology. The dyadic filter bank theory mentioned in^13^satisfies the interruption response faster than conventional system, but calculation with respect to the parameters is quite longer than usual. The^18,19,22^&^23^ are not validated experimentally, but tested in simulation platform verifying its response to fault and adaptive nature. Nevertheless, the reconfigurable protection strategy for multi-DC topologies, utilizing advanced graph algorithms, remains untested.

**Table 10:** Comparison of existing benchmark system in DC microgrid.

Reference	Paper highlights	Multi -DC microgrid topology	Rapid response to fault	Time taken for fault detection (> 0.5 ms)	Adaptive	Communication interfaced	Experimental setup validation
^11^	Advanced OC protection using K-means Clustering	**✗**	✔	✔	✔	✔	✔
^12^	IED based communication	**✗**	✔	✔	✔	✔	✔
^13^	Dyadic filter bank theory employed for DWT coefficient	**✗**	✔	✔	✔	**✗**	✔
^18^	Hybrid Passive-Overcurrent Relay for LVDC	**✗**	✔	✔	**✗**	✔	**✗**
^19,20^	Adaptive OC using fault clustering	**✗**	**✗**	✔	✔	**✗**	**✗**
^21^	Adaptive scheme based on Fano factor	**✗**	✔	✔	✔	**✗**	✔
^22^	FGI based method	**✗**	✔	✔	✔	**✗**	**✗**
^23^	EKF based method	**✗**	✔	✔	✔	**✗**	**✗**
^25^	Decentralized model based fault detection and Isolation	**✗**	✔	✔	**✗**	**✗**	✔
^26^	Ensemble classifier-based protection scheme	**✗**	✔	✔	✔	**✗**	**✗**
^27^	New fault detection and relay coordination	**✗**	✔	✔	✔	**✗**	**✗**
^28^	deep learning algorithm-based protection	**✗**	✔	✔	✔	✔	**✗**
^29^	Ground fault detection using sequentially switched grounding connections	**✗**	✔	✔	**✗**	**✗**	✔
^30^	Based on Support Vector Machines and Bagged Trees in the Machine Learning	**✗**	✔	✔	✔	**✗**	**✗**
^31^	Active Islanding Detection Method in DC Microgrids Using Real-Time Wavelet Analysis	**✗**	✔	✔	**✗**	**✗**	✔
^32^	Methodology based on injecting high-frequency oscillations for detecting high-impedance fault.	**✗**	✔	✔	✔	**✗**	**✗**
proposed	Enhanced OC protection using LOTT and Bidirectional Dial’s	✔	✔	✔	✔	✔	✔

**Table 11:** Comparison of existing with proposed algorithm for OB, DB and minimal distance path identification.

ALGORITHM	DC MICROGRID [5]			QUINTUPLE DC MICROGRID (Proposed)		
	TESTED NETWORK	PROCESSOR DEPLOYED	FAULT DETECTION TIME (ms)	TESTED NETWORK	PROCESSOR DEPLOYED	FAULT DETECTION TIME (ms)
Prims & Dijkstra	7 Bus	Intel i5	**1.87**	35 Bus	Intel i5	**7.31**
Kruskal & Floyd Warshall	7 Bus	Intel i5	**1.79**	35 Bus	Intel i5	**12.22**
Fenwick & Bidirectional Dijkstra	7 Bus	Intel i5	**0.38**	35 Bus	Intel i5	**2.88**
**LOTT & Bidirectional Dial’s (proposed)**	**7 Bus**	**Intel i5**	**0.22**	**35 Bus**	**Intel i5**	**2.64**

**Fig. 19:**
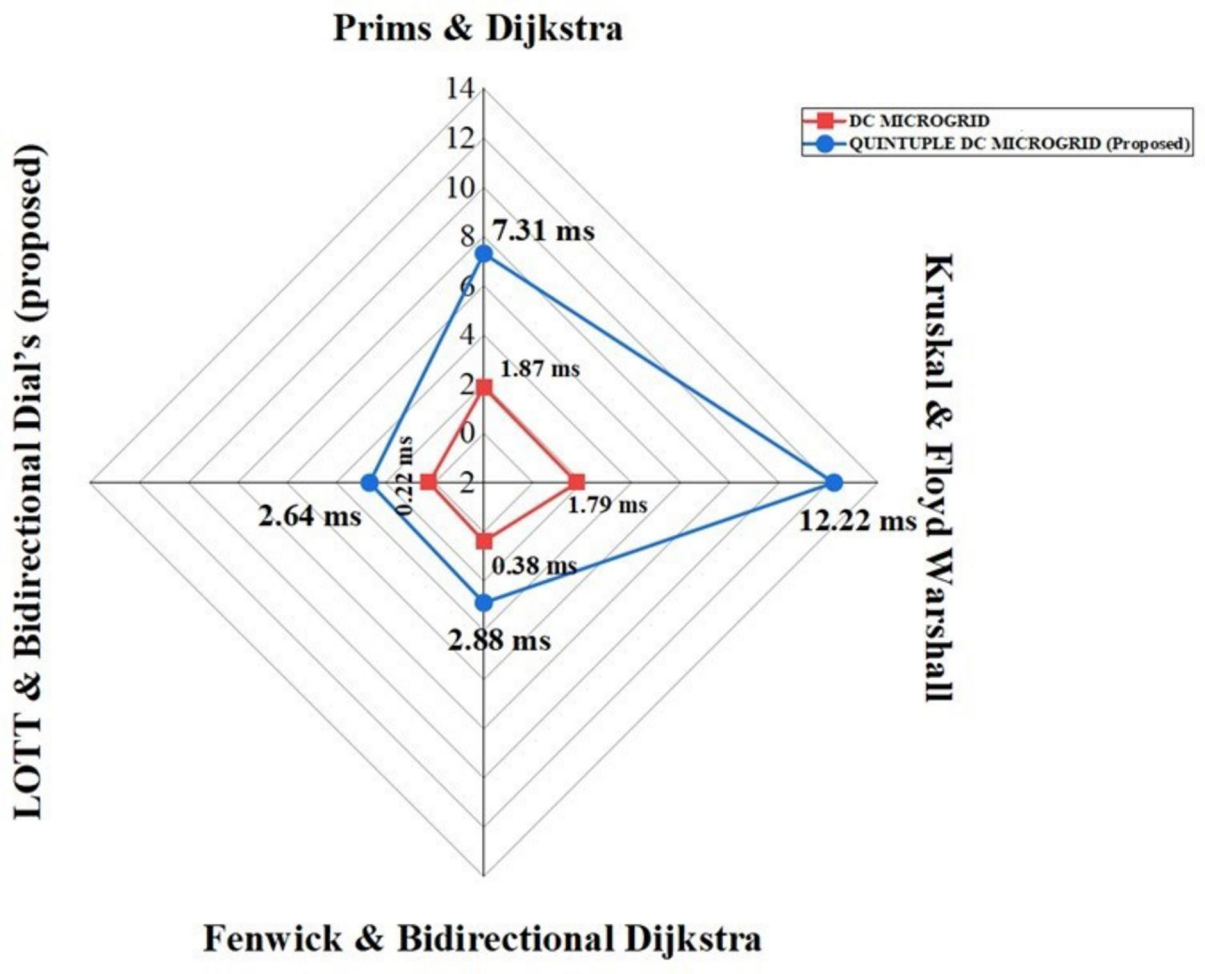
Comparison of proposed with existing algorithm for OB, DB and shortest path identification.

In the comparative analysis as shown in Table 11 between the existing algorithm and the proposed one, the run time of the quintuple DC bus system is evaluated using Intel i5 processor. Analysing the run time with real time simulator is an important step in the algorithm’s validation process as it aids in making informed decisions regarding its potential real-world application and scalability. The analysis carried with Fenwick and bidirectional dijkstra takes higher disk space even though the algorithm is included with flash program for every fifteen minutes. On comparison to the existing algorithm, the proposed works rapidly with flash program and the evaluation of fault interrupting time provides valuable insights into the algorithm’s efficiency and suitability for practical implementation as it proves the least time of 2.64 ms to interrupt the fault compared to existing algorithms. Additionally, the comparison aids in determining if the proposed algorithm offers significant improvements over the existing one in terms of execution speed and computational requirements and from the comparison with existing algorithms as shown in Fig. 19, it is understood that the combination of LOTT with bidirectional Dial’s proves its efficiency in interrupting and restoring the fault rapidly. The proposed method differs from existing approaches by offering superior adaptability to real-time applications through its integration with the Microgrid Monitoring Fault Detection System (MGMFDS), which enables the bidirectional grid to become self-healing adaptive network. The advanced data structure algorithm used in proposed protection methodology allows for rapid detection and interruption of faults, even in large networks, by reducing both time and space complexity. This method should be implemented in practice because it prevents common issues such as protection blinding and false tripping in DC microgrids, which are significant concerns with other protection systems. These advantages make our approach highly effective for improving the reliability and efficiency of microgrid operations.

## Conclusion

Designing an adaptive grid resilient scheme (AGRS) with fault identification method is a challenging task considering its grid dynamics and bidirectional power flow in microgrid. As the conventional protection scheme takes prolonged arc interruption, this article proposes a rapid fault detection and interruption technique using an advanced two novel graph algorithm “level order tree traversal (LOTT) with segment tree functions and Bidirectional Dial’s algorithm” where a grid monitoring system is made as MGMFDS integrated with SPR for rapid fault interruption. The proposed technique implemented with LOTT algorithm determines the OB and DB as it has an enhanced feature that accumulates data from segments in the network. The relay threshold datas from SPR helps the LOTT to easily predict the OB and DB, thereby the fault location can be predicted easily and the minimal distance path from fault location in close proximity to DG is identified by bidirectional Dial’s algorithm. Simultaneous fault and multi-fault scenarios are verified using the proposed algorithm to identify and interrupt all kinds of faults in DC microgrid configurations. During this analysis, LOTT with bidirectional Dial’s works rapidly in separated seven bus system with an efficiency of 99.2% and 35 bus system with an efficiency of 99.8%. As future scope of the work, the proposed adaptive protection strategies can be implemented with IoT that works in any smart automation system. This research work was carried out in Real Time simulator platform in Smart Grid Laboratory at Vellore institute of Technology.

## Electronic supplementary material

Below is the link to the electronic supplementary material.

Supplementary Material 1

## Data Availability

The datasets generated and/or analysed during the current study are available from the corresponding author on reasonable request.

## Variables

I_PV_: PV module current.
$\:\beta\:$: Rotor Pitch adjustment
I_o_: Saturation current in PV module.
N_S_, N_p_: Numeral cells in series and parallel
Rs: Resistance in series.
Pω: Power generated by wind.
ρ: Density of air.
Ar: Area swept by wind blades.
V: Wind velocity.
Cp: Coefficient of power.
S: Area swept by blade.
λ: Tip speed ratio.
V_ibb_ and i_ibb_: Voltage and current of Battery system.

## Acronyms

AGRS: Adaptive Grid Resilient System.
MGMFDS: Microgrid monitoring fault detection system.
LOTT: Level order tree traversal.
RES: Renewable Energy System.
DCCB: DC circuit breaker.
PCC: Point of Common coupling.
TMS: Time setting multiplier.
OC: Over current.
DCMG: DC Microgrid.
SPR: Smart power relay.
LUT: Look up table.
OB: Operating bus.
DB: Dormant bus.
L-L: Line - Line fault.
L-G: Line - Ground fault.
MF: Multi-fault.
SF: Simultaneous fault.
RF: Reverse fault.
PV: Photovoltaic.
PMSG: Permanent Magnet Synchronous Generator

## References

[1] Alam, M. S., Al-Ismail, F. S., Rahman, S. M., Shafiullah, M. & Md Alamgir Hossain Planning and protection of DC microgrid: a critical review on recent developments. *Eng. Sci. Technol. Int. J.* **41**10.1016/j.jestch.2023.101404 (2023). 101404, ISSN 2215 – 0986.

[2] Hosseini, S. A., Taheri, B., Sadeghi, S. H. H. & Nasiri, A. An overview of DC Microgrid Protection Schemes and the factors involved. *Electr. Power Compon. Syst.* 1–31. 10.1080/15325008.2023.2251469 (2023).

[3] Faazila, S., Fathima & Premalatha, L. Protection Strategies for AC and DC Microgrid – A Review of Protection methods adopted in recent decade. *IETE J. Res.*10.1080/03772063.2021.1990140 (2021).

[4] Gnana Swathika, O. V. & Hemamalini, S. Prims-aided Dijkstra Algorithm for Adaptive Protection in Microgrids. *IEEE J. Emerg. Sel. Top. Power Electron.* **4**, 1279–1286. 10.1109/JESTPE.2016.2581986 (2016).

[5] Faazila Fathima, S. & Premalatha L., Prithviraj Yuvaraj An advanced graph algorithm-based protection strategy for detecting kilometric and cross-country faults in DC microgrid. *Heliyon* **10**, 12, e32845. 10.1016/j.heliyon.2024.e32845 (2024). 38975111 10.1016/j.heliyon.2024.e32845 PMC11225844

[6] V, G., Malini, H., Ashre, T. & Haritha, & Chazelle-Dijkstra method using fibonacci heaps algorithm for identifying shortest path in microgrids. *Int. J. Simul. Syst. Sci. Technol.* **17** (41), 211–215. 10.5013/IJSSST.a.17.41.21 (2017).

[7] Gnana Swathika, O. V. & Hemamalini, S. *Prims Aided Floyd Warshall Algorithm for Shortest Path Identification in Microgrid, Emerging Trends in Electrical, Communications and Information Technologies*394 (Springer, 2017). Lecture Notes in Electrical Engineering10.1007/978-981-10-1540-3_30

[8] Saluja, J., Biswas, S., Roy, S. & Swathika, O. G. Performance analysis of Graph algorithms for Microgrid Protection. *J. Telecommunication Electron. Comput. Eng. (JTEC)*. **10** (1–8), 115–118 (2018).

[9] Arya, R., Yadav, R., Agarwal, R., Swathika, O. V. & & & Dijkstra’s algorithm for shortest path identification in reconfigurable microgrid. *J. Eng. Appl. Sci.* **13**, 717–720. 10.3923/jeasci.2018.717.720 (2018).

[10] Malini, H. & V, G. Graph theory and optimization algorithms aided adaptive Protection in Reconfigurable Microgrid. *J. Electr. Eng. Technol.* **15** (1), 421–431. 10.1007/s42835-019-00197-8 (2020).

[11] Sanati, S., Mosayebi, A. & Kamwa, I. Advanced Rapid Directional Over-current Protection for DC Microgrids using K-Means clustering, IEEE transactions on Power Delivery. PP. 1–12. (2024). 10.1109/TPWRD.2024.3353109

[12] Kant Kamal, A., Salauddin, G. & Hari, O. ,‘An advanced short-circuit protection scheme for a bipolar DC microgrid’ frontiers in Energy Research, **11**, ISSN 2296–598X, (2023). 10.3389/fenrg.2023.1100789

[13] Bayati, N. et al. Z 2022 EMD/HT based local fault detection in DC microgrid clusters. *IET Smart Grid* **5** 177–188, 10.1049/stg2.12060

[14] Shabani & Mazlumi, K. Evaluation of a communication-assisted Overcurrent Protection Scheme for Photovoltaic-based DC Microgrid. *IEEE Trans. Smart Grid*. **11** (1), 429–439. 10.1109/TSG.2019.2923769 (2020).

[15] Haritha, P. S., Sambhu, S. & Ravikumar Pandi, V. Communication Assisted Coordinated Protection Scheme for DC Microgrid, 2018 3rd IEEE International Conference on Recent Trends in Electronics, Information & Communication Technology (RTEICT), Bangalore, India, pp. 351–356, (2018). 10.1109/RTEICT42901.2018.9012124

[16] Pandiarajan, N. & Muthu R. Mathematical modelling of photovoltaic module with Simulink. *1st Int. Conf. Electr. Energy Syst. ICEES*, 258–263. (2011).

[17] Yehia, D. M. & Mansour, D. A. Modeling and analysis of superconducting fault current limiter for system integration of battery banks. *IEEE Trans. Appl. Supercond 5603006*. 10.1109/TASC.2018.2814398 (2018).

[18] Saleh, K. A., Hooshyar, A. & El-Saadany, E. F. Hybrid Passive-Overcurrent Relay for detection of faults in low-voltage DC Grids. *IEEE Trans. Smart Grid*. **8** (3), 1129–1138. 10.1109/TSG.2015.2477482 (2017).

[19] Saeed Sanati, M. A. Ahmed Awad, An adaptive Overcurrent Protection for Solar-based DC Microgrids using IEC 61850, arXiv:2307.01940, (2023). 10.48550/arXiv.2307.01940

[20] Ojaghi, M. & Mohammadi, V. Use of clustering to reduce the number of different setting groups for adaptive coordination of Overcurrent Relays. *IEEE Trans. Power Delivery*. **33** (3), 1204–1212. 10.1109/TPWRD.2017.2749321 (2018).

[21] Srivastava, C. & Tripathy, M. Novel adaptive Fault Detection Strategy in DC Microgrid utilizing statistical-based method. *IEEE Trans. Industr. Inf.* **19** (5), 6917–6929. 10.1109/TII.2022.3199942 (2023).

[22] Liu, H. et al. Fuzzy granulation interval-based Fault diagnosis Method for Ring-Type DC Microgrid. IEEE transactions on Smart Grid. PP. 1–1. (2024). 10.1109/TSG.2024.3359635

[23] Larik, N. A., Li, M. S. & Wu, Q. H. Enhanced Fault detection and localization strategy for high-speed protection in medium-voltage DC distribution networks using extended Kalman Filtering Algorithm, in IEEE Access, 12, pp. 30329–30344, (2024). 10.1109/ACCESS.2024.3369418

[24] J MA, V S. Implementation and proficiency analysis of enhanced graph algorithm for DC microgrid applications. *Sci. Rep.* ;**14**(1):14476. 10.1038/s41598-024-65225-8. (2024). PMID: 38914591; PMCID: PMC11196278. 10.1038/s41598-024-65225-8 PMC11196278 38914591

[25] Wang, T., Liu, W. & Hao, Z. A Decentralized Model-Based Fault Detection and Isolation Scheme for MVDC Shipboard Power Systems, in IEEE Transactions on Transportation Electrification, vol. 10, no. 4, pp. 7804–7815, Dec. (2024). 10.1109/TTE.2024.3468030

[26] Tiwari, S. P. & Koley, E. Subhojit Ghosh,Communication-less ensemble classifier-based protection scheme for DC microgrid with adaptiveness to network reconfiguration and weather intermittency, Sustainable Energy, Grids and Networks, Volume 26,2021,100460,ISSN 2352–4677,10.1016/j.segan.2021.100460

[27] Anu Bhalla, Bhavesh, R. Bhalja,a new fault detection and relay coordination technique for low voltage DC microgrid, Electric Power Systems Research, **236**, 110907, ISSN 0378–7796 (2024). 10.1016/j.epsr.2024.110907

[28] Tiwari, S. P. An efficient protection scheme for critical fault detection in microgrid under uncertain scenarios using deep learning algorithm. *Electr. Eng.*10.1007/s00202-024-02861-3 (2024).

[29] Guerrero, J. M. et al. Ground Fault detector for DC Microgrids using sequentially-switched grounding connections. *IEEE Trans. Ind. Appl.* **60** (6), 8888–8900. 10.1109/TIA.2024.3446749 (2024).

[30] Ibrahim, M. H., Badran, E. A. & Abdel-Rahman, M. H. Detect, Classify, and Locate Faults in DC Microgrids Based on Support Vector Machines and Bagged Trees in the Machine Learning Approach, in IEEE Access, vol. 12, pp. 139199–139224, (2024). 10.1109/ACCESS.2024.3466652

[31] Kazeminia, E., Goudarzitaemeh, S., Eren, S. & Bakhshai, A. Modeling and Stability Analysis of an active islanding detection method in DC Microgrids using real-time Wavelet Analysis, in IEEE Access, **12**, pp. 53767–53784, (2024). 10.1109/ACCESS.2024.3387909

[32] Srivastava, C. & Tripathy, M. Novel Grounding and Protection Strategy for DC Microgrid Restraining Fault Current, in IEEE Transactions on Power Delivery, vol. 39, no. 4, pp. 2182–2193, Aug. (2024). 10.1109/TPWRD.2024.3397711

